# Supplementary material for: Estimating the additive genetic variance for relative fitness from changes in allele frequency
Source: Genetics. 2025 Oct 28;232(1):iyaf232. doi: 10.1093/genetics/iyaf232 (PMC12774835; doi:10.1093/genetics/iyaf232)
Supplement: iyaf232_Supplementary_Data [file iyaf232_supplementary_data.pdf]

Supplementary information for 'Estimating the additive genetic variance for relative fitness from changes in allele frequency'

Manas Geeta Arun[1,*], Aidan Angus-Henry[1,2], Darren J. Obbard[1] and Jarrod D. Hadfield[1]
[1]Institute of Ecology and Evolution, The University of Edinburgh, Ashworth Laboratories Charlotte Auerbach Road, Edinburgh, EH9 3FL, United Kingdom.
[2]Charité - Universitätsmedizin Berlin, Charitéplatz 1, 10117 Berlin, Germany.
[*]Corresponding author: manas.geetaarun@ed.ac.uk

S1. The dynamics of $\mathbf{L}$ under drift and recombination.

As outlined in the section 'Extending our approach to practical situations' our goal is to infer the additive genetic variance for relative fitness in the base population ($V_{\bar{A}}(0)$) using genome-wide allele frequency changes between generations t and τ. In order to derive the expected allele frequency change due to selection over this time period, we first derive the expected dynamics of $\mathbf{L}$ under drift and recombination.

The matrix $\mathbf{L}$ is a covariance matrix whose elements are proportional to the genotypic linkage-disequilbria (off-diagonals) or variance in genotypic allele frequencies (diagonals) at the start of a generation, before selection has acted. We can decompose $\mathbf{L}$ into $\mathbf{L}^{'}$ and $\mathbf{L}^{''}$ following the notation of Buffalo and Coop (2019) where $\mathbf{L}^{'}$ represents the (co)variances that arise due to alleles in the same gamete and $\mathbf{L}^{''}$ represents the (co)variances that arise due to alleles in the different gametic contributions of a genotype. Note that in Santiago and Caballero (1998) the primes have a subtly different meaning after the initial generation, as the double prime in following generations designates gametic phase disequilibrium due to recombination and nongametic phase disequilibrium in the initial generation. The elements of $\mathbf{L}^{'}$ and $\mathbf{L}^{''}$ have direct correspondences with genetic diversities and additive measures of disequilibria. Under the notation of Weir and Cockerham (1989) (see also Bulmer (1980), Chapter 12), the diagonal elements of $\mathbf{L}^{'}$ are half the gametic genetic diversities ($\pi_i = p_i(1-p_i) = 2L^{'}_{ii}$), and the off-diagonals are half the gametic-phase disequilibria ($D_{ij} = 2L^{'}_{ij}$). Note that Weir and Cockerham (1989) uses π to denote $p_i(1-p_i)$ rather than the more usual (and less natural) $2p_i(1-p_i)$. The diagonal elements of $\mathbf{L}^{''}$ are half the additive coefficients of Hardy Weinberg disequilibria ($D_i = 2L^{''}_{ii}$), and the off-diagonals are half the nongametic-phase disequilibria ($D_{i/j} = 2L^{''}_{ij}$).

To see these correspondences, imagine two bi-allelic loci, i and j, with reference/alternate alleles A/a and B/b, respectively. There are four possible gametic haplotypes, and we can denote the frequency of haplotype AB as p_{AB} in the gametes and the frequency of allele A as p_A. The proportion of copies of the reference allele at locus i in a randomly chosen individual, c_i, can be decomposed into into the sum of maternal and paternal contribution: $c_i = m_i + f_i$ where m_i (or f_i) takes the value 1/2 if the mother (or the father) contributed a reference allele and 0 if not. Then,

$$\begin{aligned} L_{ij} &= COV(c_i, c_j) \\ &= COV(m_i + f_i, m_j + f_j) \\ &= COV(m_i, m_j) + COV(f_i, f_j) + COV(m_i, f_j) + COV(f_i, m_j) \end{aligned} \tag{S1}$$

Assuming haplotype frequencies are identical in male and female gametes we get

$$\begin{aligned} L_{ij} &= (p_{AB} - p_A p_B)/4 + (p_{AB} - p_A p_B)/4 + (p_{A/B} - p_A p_B)/4 + (p_{A/B} - p_A p_B)/4 \\ &= (p_{AB} - p_A p_B)/2 + (p_{A/B} - p_A p_B)/2 \\ &= D_{ij}/2 + D_{i/j}/2 \end{aligned} \tag{S2}$$

where $p_{A/B}$ is the frequency of zygotes that have an A from their mother and a B from their father, or vice versa. When $i = j$,

$$\begin{aligned} L_{ii} &= COV(c_i, c_i) \\ &= (p_A^2 - p_A)/2 + (p_{A/A} - p_A^2)/2 \\ &= \pi_i/2 + D_i/2 \end{aligned} \tag{S3}$$

where $p_{A/A}$ is the frequency of A homozygotes. The term D_i is an alternative, additive, measure of deviation from Hardy-Weinberg Equilibrium than the more commonly used inbreeding coefficient, F.

To derive expressions for the dynamics of $\mathbf{L}^{'}$ and $\mathbf{L}^{''}$, note that $\mathbf{L}^{''}$ is generated anew each generation and under random mating has zero expectation such that

$$L^{''}_{t+1,ij} = e^{''}_{t+1,ij} \tag{S4}$$

where $e^{''}_{t+1,ij}$ is a stochastic term with mean zero and variance given in Weir (1996). The elements of $\mathbf{L}^{'}$ under recombination and drift are (Hill and Robertson 1968; Santiago and Caballero 1998)

$$\begin{aligned} L^{'}_{t+1,ij} &= \left(1-\tfrac{1}{2N_{e_t}}\right)\left((1-r_{t,ij})L^{'}_{t,ij}+r_{t,ij}L^{''}_{t,ij}\right)+e^{'}_{t+1,ij} \\ &= z_{t,ij}L^{'}_{t,ij}+\zeta_{t,ij}L^{''}_{t,ij}+e^{'}_{t+1,ij} \end{aligned} \tag{S5}$$

where $z_{t,ij}=(1-r_{t,ij})(1-\frac{1}{2N_{e_t}})$ and $\zeta_{t,ij}=r_{t,ij}(1-\frac{1}{2N_{e_t}})$, with $r_{t,ij}$ being the recombination rate between locus i and j in generation t, and N_{e_t} the effective population size in generation t. In the absence of selection, the stochastic terms, $e^{'}_{t+1,ij}$, have zero mean and are uncorrelated over time, with variances given by Ohta and Kimura (1969, Eq 25, although those variances must be divided by 4 here since we are working with the frequency of alleles in individuals rather than gametes). The covariances between, for example, $e^{'}_{t+1,ij}$ and $e^{'}_{t+1,kl}$ are not given in Ohta and Kimura (1969), and may be unknown. Using the above results we can derive a recursion for $L^{'}$ at some arbitrary time t:

$$\begin{aligned}
L^{'}_{1,ij} &= z_{0,ij}L^{'}_{0,ij}+\zeta_{0,ij}L^{''}_{0,ij}+e^{'}_{1,ij} \\
L^{'}_{2,ij} &= z_{1,ij}(z_{0,ij}L^{'}_{0,ij}+\zeta_{0,ij}L^{''}_{0,ij}+e^{'}_{1,ij})+\zeta_{1,ij}L^{''}_{1,ij}+e^{'}_{2,ij} \\
L^{'}_{3,ij} &= z_{2,ij}(z_{1,ij}(z_{0,ij}L^{'}_{0,ij}+\zeta_{0,ij}L^{''}_{0,ij}+e^{'}_{1,ij})+\zeta_{1,ij}L^{''}_{1,ij}+e^{'}_{2,ij})+\zeta_{2,ij}L^{''}_{2,ij}+e^{'}_{3,ij} \\
L^{'}_{3,ij} &= z_{2,ij}z_{1,ij}z_{0,ij}L^{'}_{0,ij}+z_{2,ij}z_{1,ij}\zeta_{0,ij}L^{''}_{0,ij}+z_{2,ij}z_{1,ij}e^{'}_{1,ij}+z_{2,ij}\zeta_{1,ij}L^{''}_{1,ij}+z_{2,ij}e^{'}_{2,ij}+\zeta_{2,ij}L^{''}_{2,ij}+e^{'}_{3,ij} \\
\cdots &= \cdots \\
L^{'}_{t,ij} &= L^{'}_{0,ij}\prod_{k=0}^{t-1}z_{k,ij}+\sum_{k=0}^{t-1}\zeta_{k,ij}L^{''}_{k,ij}\prod_{u=k+1}^{t-1}z_{u,ij}+\sum_{k=1}^{t-1}e^{'}_{k,ij}\prod_{u=k}^{t-1}z_{u,ij}+e^{'}_{t,ij} \\
L^{'}_{t,ij} &= L^{'}_{0,ij}\prod_{k=0}^{t-1}z_{k,ij}+\zeta_{0,ij}L^{''}_{0,ij}\prod_{k=1}^{t-1}z_{k,ij}+\sum_{k=1}^{t-1}\zeta_{k,ij}L^{''}_{k,ij}\prod_{u=k+1}^{t-1}z_{u,ij}+\sum_{k=1}^{t-1}e^{'}_{k,ij}\prod_{u=k}^{t-1}z_{u,ij}+e^{'}_{t,ij} \\
L^{'}_{t,ij} &= L^{'}_{0,ij}\prod_{k=0}^{t-1}z_{k,ij}+\frac{\zeta_{0,ij}}{z_{0,ij}}L^{''}_{0,ij}\prod_{k=0}^{t-1}z_{k,ij}+\sum_{k=1}^{t-1}\frac{\zeta_{k,ij}}{z_{k,ij}}L^{''}_{k,ij}\prod_{u=k}^{t-1}z_{u,ij}+\sum_{k=1}^{t-1}e^{'}_{k,ij}\prod_{u=k}^{t-1}z_{u,ij}+e^{'}_{t,ij} \\
L^{'}_{t,ij} &= \left(L^{'}_{0,ij}+\frac{\zeta_{0,ij}}{z_{0,ij}}L^{''}_{0,ij}\right)\prod_{k=0}^{t-1}z_{k,ij}+\sum_{k=1}^{t-1}\left(e^{'}_{k,ij}+\frac{\zeta_{k,ij}}{z_{k,ij}}L^{''}_{k,ij}\right)\prod_{u=k}^{t-1}z_{u,ij}+e^{'}_{t,ij}
\end{aligned} \tag{S6}$$

Having the matrix $\mathbf{N}_t$ with the ij^{th} element $\prod_{k=0}^{t-1}z_{k,ij}$, we have for $t>0$

$$\mathbf{L}_t=\mathbf{N}_t\circ\tilde{\mathbf{L}}_0+\Delta\mathbf{L}^{'}_t+\mathbf{L}^{''}_t \tag{S7}$$

where $\circ$ is the Hadamard (i.e. lement-wise) product. The first term is the expected $\mathbf{L}$ (and $\mathbf{L}^{'}$) in generation t, conditional on the genotypic composition of the population in generation 0, where $\tilde{\mathbf{L}}_0$ is the sum of $\mathbf{L}^{'}_0$ and $\mathbf{L}^{''}_0$ with the elements of the latter weighted by $\zeta_{0,ij}/z_{0,ij}=r_{0,ij}/(1-r_{0,ij})$. $\Delta\mathbf{L}^{'}_t$ is a matrix with elements equal to the sum of the stochastic terms involving e in Equation S6 and represents stochastic changes in $\mathbf{L}^{'}$ from generation 0 to generation t. $\mathbf{L}^{''}_t$ are the new nongametic-phase disequilibria that arise in generation t. Note that if replicate populations are initiated from the offspring of Generation 0, then the stochastic terms, $\Delta\mathbf{L}^{'}_t$ and $\mathbf{L}^{''}_t$, will be unique in each replicate although the deterministic part is shared. If recombination rates are constant in time $r_{t,ij}=r_{ij}$ then $\zeta_{0,ij}/z_{0,ij}=r_{ij}/(1-r_{ij})$ and $\prod_{k=0}^{t-1}z_{k,ij}=(1-r_{ij})^t\prod_{k=0}^{t-1}(1-\frac{1}{2N_{e_k}})$. When population sizes are also constant then $\prod_{k=0}^{t-1}z_{k,ij}=(1-r_{ij})^t(1-\frac{1}{2N_e})^t$.

S2. The mean and (co)variance of allele frequency changes.

Given our model for the dynamics of $\mathbf{L}$ (See Supplementary information S1) we can derive the mean and (co)variance of the allele-frequency changes within a replicate. Here expectations and variances are taken with respect to the evolutionary process, which not only includes the effects of drift ($\underset{D}{\Delta}\mathbf{p}$) but also stochastic changes in $\mathbf{L}$ and $\boldsymbol{\alpha}$. The change in allele frequency in replicate m is

$$\begin{aligned} \Delta\mathbf{p}_m &= \sum_{t=t_m}^{\tau_m-1}\left(\mathbf{L}_t\boldsymbol{\alpha}+\underset{D}{\Delta}\mathbf{p}_{t,m}\right) \\ &= \sum_{t=t_m}^{\tau_m-1}\left((\mathbf{N}_{t,m}\circ\tilde{\mathbf{L}}_0)\bar{\boldsymbol{\alpha}}+\Delta\mathbf{L}_{t,m}\bar{\boldsymbol{\alpha}}+(\mathbf{N}_{t,m}\circ\tilde{\mathbf{L}}_0)\Delta\boldsymbol{\alpha}_{t,m}+\Delta\mathbf{L}_{t,m}\Delta\boldsymbol{\alpha}_{t,m}+\underset{D}{\Delta}\mathbf{p}_{t,m}\right) \end{aligned} \tag{S8}$$

where $\Delta\mathbf{L}_{t,m} = \Delta\mathbf{L}^{'}_{t,m} + \mathbf{L}^{''}_{t,m}$, $\bar{\boldsymbol{\alpha}}$ is a vector of mean average effects and $\Delta\boldsymbol{\alpha}_{t,m}$ a vector of deviations specific to time t and replicate m. By definition, drift is non-directional and so $E\left[\underset{D}{\Delta}\mathbf{p}_{t,m}\right] = \mathbf{0}$. We also assume that the unpredictable response to selection has zero expectation which requires that deviations in $\mathbf{L}$ and $\boldsymbol{\alpha}$ from their predicted means are zero on average ($E[\Delta\mathbf{L}] = \mathbf{0}$ and $E[\Delta\boldsymbol{\alpha}] = \mathbf{0}$) and there is no correlation between them ($COV(\Delta\mathbf{L}, \Delta\boldsymbol{\alpha}) = 0$). If the genetic architecture of fitness is sufficiently polygenic, selection-induced changes in $\mathbf{L}$ may be negligible. Similarly, since the expected change in $\mathbf{L}$ is slow, the induced change in $\boldsymbol{\alpha}$ in the presence of non-additive gene action should be minor. Making these assumptions we have:

$$
\begin{aligned}
E\left[\Delta\mathbf{p}_m\right] =& \sum_{t=t_m}^{\tau_m-1}(\mathbf{N}_{t,m}\circ\tilde{\mathbf{L}}_0)\bar{\boldsymbol{\alpha}} \\
=& \left(\tilde{\mathbf{L}}_0\circ\sum_{t=t_m}^{\tau_m-1}\mathbf{N}_{t,m}\right)\bar{\boldsymbol{\alpha}} \\
=& \left(\tilde{\mathbf{L}}_0\circ\mathbf{N}^{(m)}\right)\bar{\boldsymbol{\alpha}} \\
=& \boldsymbol{\mathcal{L}}_m\bar{\boldsymbol{\alpha}}
\end{aligned}
\tag{S9}
$$

where $\mathbf{N}^{(m)} = \sum_{t=t_m}^{\tau_m-1}\mathbf{N}_{t,m}$ and $\boldsymbol{\mathcal{L}}_m = \tilde{\mathbf{L}}_0\circ\mathbf{N}^{(m)}$. The within-replicate (co)variances are more challenging to derive. The variance due to the unpredictable selection at time t in replicate m is

$$
\begin{aligned}
VAR\left(\underset{U}{\Delta}\mathbf{p}_{t,m}\right) =& VAR\left((\Delta\mathbf{L}^{'}_{t,m}+\mathbf{L}^{''}_{t,m})\bar{\boldsymbol{\alpha}} + \boldsymbol{\mathcal{L}}_{t,m}\Delta\boldsymbol{\alpha}_{t,m} + (\Delta\mathbf{L}^{'}_{t,m}+\mathbf{L}^{''}_{t,m})\Delta\boldsymbol{\alpha}_{t,m}\right) \\
&= VAR(\Delta\mathbf{L}^{'}_{t,m}+\mathbf{L}^{''}_{t,m})\bar{\boldsymbol{\alpha}}\bar{\boldsymbol{\alpha}}^{\top} + \boldsymbol{\mathcal{L}}_{t,m}VAR(\Delta\boldsymbol{\alpha}_{t,m})\boldsymbol{\mathcal{L}}_{t,m} + VAR(\Delta\mathbf{L}^{'}_{t,m}+\mathbf{L}^{''}_{t,m})VAR(\Delta\boldsymbol{\alpha}_{t,m})
\end{aligned}
\tag{S10}
$$

In addition, covariances between the unpredictable response at different time points within a replicate will be non-zero because $\Delta\mathbf{L}^{'}_{t}$ is the sum of stochastic changes from generation 1 to t and so $COV(\Delta\mathbf{L}^{'}_{t_1,m}, \Delta\mathbf{L}^{'}_{t_2,m}) = VAR(\Delta\mathbf{L}^{'}_{t_1,m})$ when $t_1 \leq t_2$, although $COV(\mathbf{L}^{''}_{t_1,m}, \mathbf{L}^{''}_{t_2,m}) = 0$. This gives

$$
COV\left(\underset{U}{\Delta}\mathbf{p}_{t_1,m}, \underset{U}{\Delta}\mathbf{p}_{t_2,m}\right) = VAR(\Delta\mathbf{L}^{'}_{t,m})\bar{\boldsymbol{\alpha}}\bar{\boldsymbol{\alpha}}^{\top}
\tag{S11}
$$

when $t_1 \leq t_2$. Consequently, the total variance in the unpredictable response to selection will be

$$
\begin{aligned}
VAR\left(\underset{U}{\Delta}\mathbf{p}_{m}\right) =& \sum_{t=t_m}^{\tau_m-1}(2(\tau_m - t)+1)VAR(\Delta\mathbf{L}^{'}_{t,m})\bar{\boldsymbol{\alpha}}\bar{\boldsymbol{\alpha}}^{\top} \\
&+\sum_{t=t_m}^{\tau_m-1}\left(VAR(\mathbf{L}^{''}_{t,m})\bar{\boldsymbol{\alpha}}\bar{\boldsymbol{\alpha}}^{\top} + \boldsymbol{\mathcal{L}}_{t,m}VAR(\Delta\boldsymbol{\alpha}_{t,m})\boldsymbol{\mathcal{L}}_{t,m} + VAR(\Delta\mathbf{L}^{'}_{t,m}+\mathbf{L}^{''}_{t,m})VAR(\Delta\boldsymbol{\alpha}_{t,m})\right)
\end{aligned}
\tag{S12}
$$

and cannot be simplified - we simply refer to it as $\boldsymbol{\mathcal{U}}_m$.

For the drift (co)variances, note that under random mating, haplotypes are drawn from a multinomial with $2N$ trials. Using AB, Ab, AB and aB to denote the *number* of each haplotypes in the gamete pool, the number of mn haplotypes has variance $2Np_{mn}(1-p_{mn})$ and the covariance in the numbers of mn and op haplotypes is $-2Np_{mn}p_{op}$ where p_{mn} is the frequency of the mn halpotype in the gamete pool (i.e the parental haplotype frequencies modified by recombination). The covariance in the number of A and B alleles sampled, is therefore

$$
\begin{aligned}
COV(AB+Ab, AB+aB) =& COV(AB,AB)+COV(AB,aB) \\
&+COV(Ab,AB)+COV(Ab,aB) \\
=& 2N\left[p_{AB}(1-p_{AB}) - p_{AB}p_{aB} - p_{Ab}p_{AB} - p_{Ab}p_{aB}\right] \\
=& 2N\left[p_{AB}(1-p_{AB}-p_{aB}) - p_{Ab}(p_{AB}+p_{aB})\right] \\
=& 2N\left[p_{AB}(1-p_{B}) - p_{Ab}p_{B}\right] \\
=& 2N\left[p_{AB} - (p_{Ab}+p_{AB})p_{B}\right] \\
=& 2N\left[p_{AB} - p_{A}p_{B}\right] \\
=& 4N\bar{L}^{'}_{ij}
\end{aligned}
\tag{S13}
$$

where $\bar{L}^{'}_{ij}$ is the gametic-phase linkage disequilibria that would be achieved in an infinite population. We can divide through by $(1/2N)^2$ to obtain the drift covariance in frequency (rather than counts) as $\bar{L}^{'}_{ij}/N$ (i.e the drift (co)variances in a allele frequency from generation t to $t+1$ are proportional to the gametic-phase disequilibria in generation $t+1$ that would be achieved in an infinite population conditional on the genotypic composition of the population in generation t). This recovers the well known result for the drift variance in allele frequency: $\bar{L}^{'}_{ii}/N = p_i(1-p_i)/2N$. We can replace the census population size, N, with the effective population size N_E, since this approximates the sampling of genotypes in non-idealised populations well (Ethier and Nagylaki 1980). However, note that N_E differs from N_e in that it does include the impact of linked selection since this is conditioned on in the expectation, $E[\Delta\mathbf{p}]$. In matrix terms (since $\bar{L}^{'}_{t+1,ij} = L^{'}_{t,ij}(1-r_{ij}) + L^{''}_{t,ij}r_{ij}$):

$$\begin{aligned}
VAR\left(\underset{D}{\Delta}\mathbf{p}_{t,m}\right) &= \frac{1}{N_{E_{t,m}}}\bar{\mathbf{L}}^{'}_{t+1,m} \\
VAR\left(\underset{D}{\Delta}\mathbf{p}_{t,m}\right) &= \frac{1}{N_{E_{t,m}}}\left[\mathbf{R}_{-}\circ\mathbf{L}^{'}_{t,m}+\mathbf{R}_{+}\circ\mathbf{L}^{''}_{t,m}\right] \\
VAR\left(\underset{D}{\Delta}\mathbf{p}_{t,m}\right) &= \frac{1}{N_{E_{t,m}}}\left[\mathbf{R}_{-}\circ\left(\mathbf{N}_{t,m}\circ\tilde{\mathbf{L}}_{0}+\Delta\mathbf{L}^{'}_{t,m}\right)+\mathbf{R}_{+}\circ\mathbf{L}^{''}_{t,m}\right] \\
VAR\left(\underset{D}{\Delta}\mathbf{p}_{t,m}\right) &= \frac{1}{N_{E_{t,m}}}\left[\mathbf{R}_{-}\circ\mathbf{N}_{t,m}\circ\tilde{\mathbf{L}}_{0}+\mathbf{R}_{-}\circ\Delta\mathbf{L}^{'}_{t,m}+\mathbf{R}_{+}\circ\mathbf{L}^{''}_{t,m}\right]
\end{aligned} \tag{S14}$$

where $\mathbf{R}_{+}$ and $\mathbf{R}\_$ are matrices with the ij^{th} elements being r_{ij} and $1-r_{ij}$ respectively. The expected drift terms (averaged over the evolutionary stochasticity in $\mathbf{L}$) is therefore:

$$E\left[VAR\left(\underset{D}{\Delta}\mathbf{p}_{t,m}\right)\right] = \frac{1}{N_{E_{t,m}}}\left(\mathbf{R}_{-}\circ\mathbf{N}_{t,m}\circ\tilde{\mathbf{L}}_{0}\right) \tag{S15}$$

We define a new matrix $\mathbf{M}_{t,m}$ with the ij^{th} element being $(1-r_{ij})/N_{E_{t,m}}$ ($\mathbf{M}_{t,m}=\frac{1}{N_{E_{t,m}}}\mathbf{R}_{-}$) to give

$$E\left[VAR\left(\underset{D}{\Delta}\mathbf{p}_{t,m}\right)\right] = \mathbf{M}_{t,m}\circ\mathbf{N}_{t,m}\circ\tilde{\mathbf{L}}_{0} \tag{S16}$$

Since the drift terms are independent, this gives:

$$\begin{aligned}
E\left[VAR\left(\underset{D}{\Delta}\mathbf{p}_{m}\right)\right] &= \textstyle\sum_{t=t_m}^{\tau_m-1}E\left[VAR\left(\underset{D}{\Delta}\mathbf{p}_{t,m}\right)\right] \\
&= \textstyle\sum_{t=t_m}^{\tau_m-1}\frac{1}{N_{E_{t,m}}}\left(\mathbf{R}_{-}\circ\mathbf{N}_{t,m}\circ\tilde{\mathbf{L}}_{0}\right) \\
&= \tilde{\mathbf{L}}_{0}\circ\textstyle\sum_{t=t_m}^{\tau_m-1}\mathbf{M}_{t,m}\circ\mathbf{N}_{t,m} \\
&= \tilde{\mathbf{L}}_{0}\circ\mathbf{M}^{(m)}
\end{aligned} \tag{S17}$$

where $\mathbf{M}^{(m)}=\sum_{t=t_m}^{\tau_m-1}\mathbf{M}_{t,m}\circ\mathbf{N}_{t,m}$ and is null when $t_m=0$ and $\tau_m=1$. Note the ij^{th} element of $\mathbf{M}_{t,m}\circ\mathbf{N}_{t,m}=(1-r_{ij})^{t+1}\frac{1}{N_{E_t}}\prod_{k=0}^{t-1}(1-\frac{1}{2N_{e_{k,m}}})$ which reduces to $(1-r_{ij})^{t+1}\frac{1}{N_{em}}(1-\frac{1}{2N_{em}})^{t}$ with constant population size and $N_e=N_E$. $\tilde{\mathbf{L}}_{0}\circ\mathbf{M}^{(m)}$ is denoted as $\boldsymbol{\mathcal{D}}_m$ in the main text. It is not clear how large the evolutionary variance in $VAR\left(\underset{D}{\Delta}\mathbf{p}_{m}\right)$ is, and whether this would need to be accommodated.

When the vector of average effects, $\boldsymbol{\alpha}$, is constant in time and across replicates, $V_A(0)=\boldsymbol{\alpha}\mathbf{L}_0\boldsymbol{\alpha}^{\top}=\bar{\boldsymbol{\alpha}}\mathbf{L}_0\bar{\boldsymbol{\alpha}}^{\top}$. However, when they do vary, this equality does not hold and so we use the notation $V_{\bar{A}}(0)$ to distinguish $\bar{\boldsymbol{\alpha}}\mathbf{L}_0\bar{\boldsymbol{\alpha}}^{\top}$ from $V_A(0)$. We claim that $V_{\bar{A}}(0)$ can be interpreted as the genetic covariance in fitness between time-replicate combinations for a population with a genotypic composition identical to the base population: it is the expected covariance in breeding value of an individual taken from the base population and (hypothetically) raised in two different replicates at two different times.

To see this, we switch from our original definition of $\boldsymbol{\alpha}_{t,m}$ as being equal to $\bar{\boldsymbol{\alpha}}+\Delta\boldsymbol{\alpha}_{t,m}$ and with some abuse of notation use the form $\bar{\boldsymbol{\alpha}}+\Delta\boldsymbol{\alpha}_{t}+\Delta\boldsymbol{\alpha}_{m}+\Delta\boldsymbol{\alpha}_{t,m}$ where $\Delta\boldsymbol{\alpha}_{t}$ is the deviation of the average effects (averaged over replicates) at time t and $\Delta\boldsymbol{\alpha}_{m}$ is the deviation of the average effects (averaged over time) in replicate m, leaving $\Delta\boldsymbol{\alpha}_{t,m}$ as the 'residual' deviation in $\boldsymbol{\alpha}$. By definition, the terms $\Delta\boldsymbol{\alpha}_{t}$, $\Delta\boldsymbol{\alpha}_{m}$ and $\Delta\boldsymbol{\alpha}_{t,m}$ have expectation zero, where the expectations are taken over time, replicates and all time-replicate combinations respectively. $C_{\alpha_{t_m},\alpha_{\tau_n}}=\boldsymbol{\alpha}_{t,m}^{\top}\mathbf{L}_0\boldsymbol{\alpha}_{\tau,n}$ is the additive genetic covariance between replicate m at time t and replicate n at time τ given the base population structure.

$$C_{\alpha_{t_m},\alpha_{\tau_n}}=\left(\bar{\boldsymbol{\alpha}}^{\top}+\Delta\boldsymbol{\alpha}_{t}^{\top}+\Delta\boldsymbol{\alpha}_{m}^{\top}+\Delta\boldsymbol{\alpha}_{t,m}^{\top}\right)\mathbf{L}_0\left(\bar{\boldsymbol{\alpha}}+\Delta\boldsymbol{\alpha}_{\tau}+\Delta\boldsymbol{\alpha}_{n}+\Delta\boldsymbol{\alpha}_{\tau,n}\right) \tag{S18}$$

If we take the expectation of this over pairs of replicates we get:

$$C_{\alpha_t,\alpha_\tau}=\left(\bar{\boldsymbol{\alpha}}^{\top}+\Delta\boldsymbol{\alpha}_{t}^{\top}\right)\mathbf{L}_0\left(\bar{\boldsymbol{\alpha}}+\Delta\boldsymbol{\alpha}_{\tau}\right) \tag{S19}$$

since the Δ terms, and the product of pairs of Δ terms when $n\neq m$, have zero expectation. Here, C_{α_t,α_τ} is the between-replicate covariance at time t and τ, and has expectation (with respect to time) $\bar{\boldsymbol{\alpha}}^{\top}\mathbf{L}_0\bar{\boldsymbol{\alpha}}$ when $t\neq\tau$ and $\bar{\boldsymbol{\alpha}}^{\top}\mathbf{L}_0\bar{\boldsymbol{\alpha}}+Tr(\mathbf{L}_0 VAR(\Delta\boldsymbol{\alpha}_t))$ when $t=\tau$ where $VAR(\Delta\boldsymbol{\alpha}_t)$ are the (co)variances of $\boldsymbol{\alpha}$ over time.

S3. Treating α as random

In standard quantitative genetic theory, $\boldsymbol{\alpha}$ is considered fixed and the genotypes of individuals in the population are considered random, leading to a distribution of breeding values with variance V_A. More recently, however, dense molecular markers have been used as covariates for predicting genetic values, and the associated coefficients have been treated as random (Meuwissen *et al.* 2001). As noted by Gianola *et al.* (2009), this raises interpretational problems when trying to relate the variance parameters associated with the coefficients to the concept of V_A.

In our inference section we show that

$$\begin{aligned} E[V_A(0)] &= E[\boldsymbol{\alpha}^\top \mathbf{L}_0 \boldsymbol{\alpha}] \\ &= Tr(\mathbf{L}_0 \mathbf{V}_\alpha) + \boldsymbol{\mu}_\alpha^\top \mathbf{L}_0 \boldsymbol{\mu}_\alpha \end{aligned} \tag{S20}$$

where here we assume the average effects are constant across time/replicates for ease and $\boldsymbol{\mu}_\alpha$ and $\mathbf{V}_\alpha$ are the mean vector and covariance matrix for the average effects (see the Appendix in Gianola *et al.* (2009) also). Critically, we interpret this expectation as an average over the epistemic uncertainty in $\boldsymbol{\alpha}$: it is a posterior mean after marginalising the distribution of $\boldsymbol{\alpha}$ (but conditioning on $\boldsymbol{\mu}_\alpha$ and $\mathbf{V}_\alpha$):

$$E[V_A(0)|\boldsymbol{\mu}_\alpha, \mathbf{V}_\alpha, \mathbf{L}_0] \propto \int_{\boldsymbol{\alpha}} \boldsymbol{\alpha}^\top \mathbf{L}_0 \boldsymbol{\alpha} \cdot |\mathbf{J}| \cdot Pr(\boldsymbol{\alpha}|\boldsymbol{\mu}_\alpha, \mathbf{V}_\alpha, \mathbf{L}_0) d\boldsymbol{\alpha} \tag{S21}$$

where $\mathbf{J}$ is the Jacobian of the transform from $\boldsymbol{\alpha}$ to V_A and depends on $\mathbf{L}_0$.

For an inferential method we see no contradiction between treating $\boldsymbol{\alpha}$ as fixed when *defining* V_A but treating it as random when *estimating* V_A. However, when treating $\bar{\boldsymbol{\alpha}}$ as random it is important to ensure that the underlying model is invariant with respect to which allele is chosen as the reference. Quantities such as V_A and genomic best linear unbiased predictors (gBLUP) are insensitive to which allele at a locus is chosen as the reference allele and which allele is chosen as the alternate allele. To make this explicit, consider the diagonal 'assignment' matrix $\mathbf{A}$ for which the diagonal elements are either 1 (the fittest allele is the reference allele) or -1 (the fittest allele is the alternate allele). Under a particular assignment, $\boldsymbol{\alpha} = \mathbf{A}\boldsymbol{\alpha}_+$ and $\mathbf{L} = \mathbf{A}\mathbf{L}_+\mathbf{A}$, where the subscript $+$ indicates the quantity had the fitter of the two alleles been the reference allele at all loci. If we consider V_A conditional on a particular assignment we get (since $\mathbf{AA} = \mathbf{I}$):

$$\begin{aligned} V_A = &\ \boldsymbol{\alpha}^\top \mathbf{L} \boldsymbol{\alpha} \\ = &\ (\mathbf{A}\boldsymbol{\alpha}_+)^\top \mathbf{A}\mathbf{L}_+\mathbf{A}\mathbf{A}\boldsymbol{\alpha}_+ \\ = &\ \boldsymbol{\alpha}_+^\top \mathbf{A}\mathbf{A}\mathbf{L}_+\mathbf{A}\mathbf{A}\boldsymbol{\alpha}_+ \\ = &\ \boldsymbol{\alpha}_+^\top \mathbf{L}_+ \boldsymbol{\alpha}_+ \end{aligned} \tag{S22}$$

showing we get the same value of V_A irrespective of the assignment we choose. In contrast, quantities such as $E[\mathbf{L}\boldsymbol{\alpha}]$ are sensitive to the assignment, since they undergo a sign reversal under a different choice:

$$\begin{aligned} \mathbf{L}\boldsymbol{\alpha} = &\ \mathbf{A}\mathbf{L}_+\mathbf{A}\mathbf{A}\boldsymbol{\alpha}_+ \\ = &\ \mathbf{A}\mathbf{L}_+\boldsymbol{\alpha}_+ \end{aligned} \tag{S23}$$

It is tempting to use the argument that $E[\mathbf{L}\boldsymbol{\alpha}] = \mathbf{0}$ when the reference allele is chosen at random, since α is equally likely to be positive as negative. The logic behind this argument can be expressed mathematically as $E[\boldsymbol{\alpha}] = E[\mathbf{A}]E[\boldsymbol{\alpha}_+]$ since the reference allele is chosen at random and so $\mathbf{A}$ must be independent of $\boldsymbol{\alpha}_+$. Under this same assumption $E[\mathbf{A}] = \mathbf{0}$, since any diagonal element has an equal chance of being -1 or 1, such that $E[\mathbf{L}\boldsymbol{\alpha}] = 0$. However, this logic is incorrect. The argument envisages $\mathbf{A}$ as random, yet for any particular analysis $\mathbf{A}$ is no longer a random variable but fixed - a choice has been made as to which allele is the reference allele - even if there remains epistemic uncertainty as to whether the reference allele is the fitter of the two alleles.

When the reference allele is chosen arbitrarily, any sensible distribution for $\boldsymbol{\alpha}$ must induce the same distribution on $\boldsymbol{\alpha}_+$ regardless of the assignment. If $\boldsymbol{\mu}_{\alpha_+}$ and $\mathbf{V}_{\alpha_+}$ are the means and (co)variances of the average effects had all reference alleles been the fitter allele, then the distribution for a particular assignment becomes $\boldsymbol{\mu}_\alpha = \mathbf{A}\boldsymbol{\mu}_{\alpha_+}$ and $\mathbf{V}_\alpha = \mathbf{A}\mathbf{V}_{\alpha_+}\mathbf{A}$. Given $\mathbf{A}^{-1} = \mathbf{A}$ this implies $\boldsymbol{\mu}_{\alpha_+} = \mathbf{A}\boldsymbol{\mu}_\alpha$ and $\mathbf{V}_{\alpha_+} = \mathbf{A}\mathbf{V}_\alpha\mathbf{A}$. For, $\boldsymbol{\mu}_\alpha$ this implies that suitable models should be (weighted) sums of differences between invariant properties of the alleles such that the difference reverses sign when the reference and alternate allele are switched. This might be their (log) frequency, such that the model is $\beta(p-q)$, with β a parameter, or it might be the difference in derived vs ancestral coded as 1 vs -1, such that the model is 2β or -2β depending on whether the reference allele is derived or ancestral, respectively.

If $\mathbf{V}_{\bar{\alpha}}$ is assumed to be diagonal, all models are permissible since the square removes any sign. Since the multiplication of diagonal matrices is not affected by order we can see this directly:

$$\begin{aligned} \mathbf{V}_{\alpha_+} &= \mathbf{A}\mathbf{V}_{\alpha}\mathbf{A} \\ \mathbf{V}_{\alpha_+} &= \mathbf{A}\mathbf{A}\mathbf{V}_{\alpha} \\ \mathbf{V}_{\alpha_+} &= \mathbf{V}_{\alpha} \end{aligned} \tag{S24}$$

However, for non-diagonal matrices a suitable distribution must result in a sign reversal of all covariances at a locus when the reference and alternate alleles are switched. The most obvious way to achieve this is to allow $\mathbf{V}_\alpha$ to be proportional to $\mathbf{L}^p$ since

$$\begin{aligned} \mathbf{L}^p &= (\mathbf{A}\mathbf{L}_+\mathbf{A})^p \\ &= \mathbf{A}\mathbf{L}_+\mathbf{A}...\mathbf{A}\mathbf{L}_+\mathbf{A} \\ &= \mathbf{A}\mathbf{L}_+^p\mathbf{A} \end{aligned} \tag{S25}$$

where $\mathbf{L}_+$ is the linkage-disequilibrium matrix had the fittest allele been the reference allele at all loci. Under this assumption

$$\begin{aligned} \mathbf{V}_{\alpha_+} &= \mathbf{A}\mathbf{V}_{\alpha}\mathbf{A} \\ \mathbf{V}_{\alpha_+} &\propto \mathbf{A}\mathbf{L}^p\mathbf{A} \\ \mathbf{V}_{\alpha_+} &\propto \mathbf{A}\mathbf{A}\mathbf{L}_+^p\mathbf{A}\mathbf{A} \\ \mathbf{V}_{\alpha_+} &\propto \mathbf{L}_+^p \end{aligned} \tag{S26}$$

A similar model was proposed by Zeng *et al.* (2018), although there, $\mathbf{L}$ was treated as diagonal such that the variance of the average effects are assumed to be proportional to the genetic diversities to some power.

S4. Projection matrices

Rather than working with original allele frequencies, we project them into a new reduced subspace defined by $\mathbf{L}_0$ and claim that $V_A(0)$ does not depend on allele frequency changes outside of this space. de Los Campos *et al.* (2015) (in Supplementary Methods I) show that additive genetic variances are rotationally invariant: they are invariant to arbitrary linear transformations of the genotypes. Have $\mathbf{U}$ be *all* eigenvectors of $\mathbf{L}_0$ and $\mathbf{D}$ a diagonal matrix with the eigenvalues of $\mathbf{L}_0$ square-rooted along the diagonal. If we use the transformation $\mathbf{U}^\top$, de Los Campos *et al.* (2015) show that the average effects in the projected space are $\mathbf{U}^{-\top}\boldsymbol{\alpha}$ and the covariance of the projected genotypes is $\mathbf{U}^\top\mathbf{L}_0\mathbf{U}$, and:

$$V_A(0) = \boldsymbol{\alpha}^\top\mathbf{U}^{-\top}\mathbf{U}^\top\mathbf{L}_0\mathbf{U}\mathbf{U}^{-1}\boldsymbol{\alpha} = \boldsymbol{\alpha}^\top\mathbf{L}_0\boldsymbol{\alpha} \tag{S27}$$

Since $\mathbf{L}_0 = \mathbf{U}\mathbf{D}\mathbf{D}\mathbf{U}^\top$,

$$V_A(0) = \boldsymbol{\alpha}^\top\mathbf{U}^{-\top}\mathbf{U}^\top\mathbf{U}\mathbf{D}\mathbf{D}\mathbf{U}^\top\mathbf{U}\mathbf{U}^{-1}\boldsymbol{\alpha} \tag{S28}$$

which reduces to

$$V_A(0) = \boldsymbol{\alpha}^\top\mathbf{U}^{-\top}\mathbf{D}\mathbf{D}\mathbf{U}^{-1}\boldsymbol{\alpha} \tag{S29}$$

since eigenvectors are unitary $\mathbf{U}^{-1} = \mathbf{U}^\top$ and so $\mathbf{U}\mathbf{U}^\top = \mathbf{I}$. The maximum rank of $\mathbf{L}_0$ will generally be the number of individuals in the base population and will be substantially less then the number of loci. Consequently some diagonal elements of $\mathbf{D}$ will be zero which will set all elements in the corresponding column of $\mathbf{U}^{-\top}\mathbf{D}$ to zero. Consequently we can simply define the projection matrix $\mathbf{U}_\mathbf{L}$ with all columns associated with zero eigenvalues dropped without loss of information.

To show that our chosen projection ($\mathbf{P} = \mathbf{D}_2^{-1}\mathbf{U}_2^\top\mathbf{U}_\mathbf{L}^\top$) results in identical and independently distributed residuals, we need to show that the drift covariance matrix is an identity matrix under this projection. If we write down the eigendecomposition of $\mathbf{U}_\mathbf{L}^\top(\tilde{\mathbf{L}}_0 \circ \mathbf{M}^{(m)})\mathbf{U}_\mathbf{L} = \mathbf{U}_2\mathbf{D}_2\mathbf{D}_2\mathbf{U}_2^\top$, and use the unitary property of $\mathbf{U}_\mathbf{L}$ to note that $\tilde{\mathbf{L}}_0 \circ \mathbf{M}^{(m)}$ can be expressed as $\mathbf{U}_\mathbf{L}\mathbf{U}_2\mathbf{D}_2\mathbf{D}_2\mathbf{U}_2^\top\mathbf{U}_\mathbf{L}^\top$. The drift covariance matrix under the projection is then:

$$\begin{aligned} VAR(\underset{D}{\Delta}\vec{\mathbf{p}}_m) &= \mathbf{P}VAR(\underset{D}{\Delta}\mathbf{p}_m)\mathbf{P}^\top \\ &= \mathbf{P}(\tilde{\mathbf{L}}_0 \circ \mathbf{M}^{(m)})\mathbf{P}^\top \\ &= \mathbf{P}\mathbf{U}_\mathbf{L}\mathbf{U}_2\mathbf{D}_2\mathbf{D}_2\mathbf{U}_2^\top\mathbf{U}_\mathbf{L}^\top\mathbf{P}^\top \\ &= \mathbf{D}_2^{-1}\mathbf{U}_2^\top\mathbf{U}_\mathbf{L}^\top\mathbf{U}_\mathbf{L}\mathbf{U}_2\mathbf{D}_2\mathbf{D}_2\mathbf{U}_2^\top\mathbf{U}_\mathbf{L}^\top\mathbf{U}_\mathbf{L}\mathbf{U}_2\mathbf{D}_2^{-1} \\ &= \mathbf{I} \end{aligned} \tag{S30}$$

S5. Bias correction for $\boldsymbol{\mu}_{\bar{\alpha}}^{\top}\mathbf{L}_0\boldsymbol{\mu}_{\bar{\alpha}}$

The expression for $E[V_{\bar{A}}(0)]$ involves a quadratic in the vector of expected average effects, $\boldsymbol{\mu}_{\bar{\alpha}}$: $\boldsymbol{\mu}_{\bar{\alpha}}^{\top}\mathbf{L}_0\boldsymbol{\mu}_{\bar{\alpha}}$. Replacing $\boldsymbol{\mu}_{\bar{\alpha}}$ with an estimate of it, $\widehat{\boldsymbol{\mu}_{\bar{\alpha}}}$, will generally result in the estimate of $V_{\bar{A}}(0)$ being upwardly biased even if $\widehat{\boldsymbol{\mu}_{\bar{\alpha}}}$ is an unbiased estimate of $\boldsymbol{\mu}_{\bar{\alpha}}$. To understand this, and correct for it, have the linear model

$$\boldsymbol{\mu}_{\bar{\alpha}} = \mathbf{X}\boldsymbol{\beta}_{\bar{\alpha}} \tag{S31}$$

where $\mathbf{X}$ is the fixed-effect design matrix and $\boldsymbol{\beta}_{\bar{\alpha}}$ an associated parameter vector. In our analyses of simulated data, $\mathbf{X}$ only has one column, $\mathbf{p}_0 - \mathbf{q}_0$, and $\boldsymbol{\beta}_{\bar{\alpha}}$ is a scalar: $\beta_{\bar{\alpha}}^{(1)}$. We can have

$$\widehat{\boldsymbol{\beta}_{\bar{\alpha}}} = \boldsymbol{\beta}_{\bar{\alpha}} + \mathbf{m}_{\bar{\alpha}} \tag{S32}$$

where $\mathbf{m}_{\bar{\alpha}}$ is the vector of deviations of the estimates from their true value. The expected estimate of $\boldsymbol{\mu}_{\bar{\alpha}}^{\top}\mathbf{L}_0\boldsymbol{\mu}_{\bar{\alpha}}$ is then

$$\begin{aligned} E[\widehat{\boldsymbol{\mu}_{\bar{\alpha}}}^{\top}\mathbf{L}_0\widehat{\boldsymbol{\mu}_{\bar{\alpha}}}] &= \boldsymbol{\beta}_{\bar{\alpha}}^{\top}\mathbf{X}^{\top}\mathbf{L}_0\mathbf{X}\boldsymbol{\beta}_{\bar{\alpha}} + E[\mathbf{m}_{\bar{\alpha}}^{\top}\mathbf{X}^{\top}\mathbf{L}_0\mathbf{X}\mathbf{m}_{\bar{\alpha}}] \\ &= \boldsymbol{\mu}_{\bar{\alpha}}^{\top}\mathbf{L}_0\boldsymbol{\mu}_{\bar{\alpha}} + E[\mathbf{m}_{\bar{\alpha}}^{\top}\mathbf{X}^{\top}\mathbf{L}_0\mathbf{X}\mathbf{m}_{\bar{\alpha}}] \end{aligned} \tag{S33}$$

Here, the expectation is taken over the distribution of estimates, and it is assumed the estimates are unbiased (since then, $E[\mathbf{m}_{\bar{\alpha}}] = \mathbf{0}$, and so $E[\mathbf{m}_{\bar{\alpha}}\boldsymbol{\beta}_{\bar{\alpha}}^{\top}] = \mathbf{0}$). This same assumption implies

$$E[\widehat{\boldsymbol{\mu}_{\bar{\alpha}}}^{\top}\mathbf{L}_0\widehat{\boldsymbol{\mu}_{\bar{\alpha}}}] = \boldsymbol{\mu}_{\bar{\alpha}}^{\top}\mathbf{L}_0\boldsymbol{\mu}_{\bar{\alpha}} + Tr\left(\mathbf{X}^{\top}\mathbf{L}_0\mathbf{X}E[\mathbf{m}_{\bar{\alpha}}^{\top}\mathbf{m}_{\bar{\alpha}}]\right) \tag{S34}$$

Since $E[\mathbf{m}_{\bar{\alpha}}^{\top}\mathbf{m}_{\bar{\alpha}}]$ is a matrix of sampling (co)variances for the parameters, to get an improved estimate we can use the inverse Hessian to get an approximate $\mathbf{S}_{\bar{\alpha}} = E[\mathbf{m}_{\bar{\alpha}}^{\top}\mathbf{m}_{\bar{\alpha}}]$:

$$\widehat{\boldsymbol{\mu}_{\bar{\alpha}}^{\top}\mathbf{L}_0\boldsymbol{\mu}_{\bar{\alpha}}} = \widehat{\boldsymbol{\mu}_{\bar{\alpha}}}^{\top}\mathbf{L}_0\widehat{\boldsymbol{\mu}_{\bar{\alpha}}} - Tr\left(\mathbf{X}^{\top}\mathbf{L}_0\mathbf{X}\mathbf{S}_{\bar{\alpha}}\right) \tag{S35}$$

In addition, $Tr(\mathbf{L}_0\widehat{\mathbf{V}_{\bar{\alpha}}})$ is most likely an upwardly biased estimator of $Tr(\mathbf{L}_0\mathbf{V}_{\bar{\alpha}})$. However, we found no easy way to determine or correct for the degree of bias, and the simulation results suggest that the bias is likely to be small, at least when the number of replicates or generations is moderately large.

S6. Analysing allele frequency data from pool-seq

The main derivation of our method assumes allele frequency changes in each replicate are known without error, and the majority of our simulations were analysed with this being true. However, this assumption is unlikely to be met in reality and here we develop a method for incorporating uncertainty in allele frequencies estimated using pool-seq.

Assuming that all individuals have the same probability of being sampled, the covariance in reference allele number at locus i and j is equal to the number of reads that span both sites, O_{ij}, multiplied by the covariance in allele number across haplotypes $2L'_{i,j}$. To get the covariance in reference allele frequency we must divide this by the number of reads spanning site i (O_{ii}) and the number of reads spanning site j to get $2L'_{i,j}O_{ij}/(O_{ii}O_{jj})$. When $i = j$ this reduces to the familiar binomial variance for allele frequency at a single site $2L'_{i,i}O_{ii}/(O_{ii}O_{ii}) = pq/O_{ii}$. When analysing binomial data the variance often exceeds its expectation - a phenomenon known as overdisperion. In the context of pool-seq this will be mostly driven by individuals, rather than gametic contributions, varying in their probability of being sampled. In this instance, the exact form of the sampling (co)variances will depend in a complicated way on both $\mathbf{L}'$ and $\mathbf{L}''$, but here we assume that for sites close enough to be spanned by the same read $\mathbf{L}'$ dominates over $\mathbf{L}''$ and at a site pq dominates over $\mathbf{L}''$. We then simply accommodate any overdispersion using a parameter that scales the sampling (co)variances, σ_o^2, which is constant over loci. This approach is widely used when analysing binomial data (the qausibinomial approach - (McCullagh and Nelder 1989)) and values greater than one indicate overdispersion. In what follows we also assume σ_o^2 is constant across replicate/time-points although this could be relaxed.

Having the matrix $\mathbf{Q}$ with the ij^{th} element equal to $O_{ij}/(O_{ii}O_{jj})$ the sampling covariance in allele frequency in replicate m at time t_m is equal to $2\sigma_o^2(\boldsymbol{L}'_{t_m,m} \circ \mathbf{Q}_{t_m,m})$. Since errors in allele frequency estimates are independent at different time points the expected sampling covariance in allele frequency change is proportional to:

$$\begin{aligned} \boldsymbol{\mathcal{E}}_m &= 2\sigma_o^2 E\left[\boldsymbol{L}'_{t_m,m} \circ \mathbf{Q}_{t_m,m} + \boldsymbol{L}'_{\tau_m,m} \circ \mathbf{Q}_{\tau_m,m}\right] \\ &= 2\sigma_o^2 E\left[(\boldsymbol{\mathcal{L}}_{t_m,m} + \Delta\boldsymbol{L}'_{t_m,m}) \circ \mathbf{Q}_{t_m,m} + (\boldsymbol{\mathcal{L}}_{\tau_m,m} + \Delta\boldsymbol{L}'_{\tau_m,m}) \circ \mathbf{Q}_{\tau_m,m}\right] \\ &= 2\sigma_o^2\left(\boldsymbol{\mathcal{L}}_{t_m,m} \circ \mathbf{Q}_{t_m,m} + \boldsymbol{\mathcal{L}}_{\tau_m,m} \circ \mathbf{Q}_{\tau_m,m}\right) \end{aligned} \tag{S36}$$

where the expectation is taken over evolutionary realisations of $\mathbf{L}'$ and depends on $E[\Delta\boldsymbol{L}'_{t,m}] = 0$. The expected sampling covariance in projected allele frequency change is therefore:

$$\overrightarrow{\boldsymbol{\mathcal{E}}}_m = 2\sigma_o^2\mathbf{P}\left(\boldsymbol{\mathcal{L}}_{t_m,m} \circ \mathbf{Q}_{t_m,m} + \boldsymbol{\mathcal{L}}_{\tau_m,m} \circ \mathbf{Q}_{\tau_m,m}\right)\mathbf{P}^{\top} \tag{S37}$$

$\overrightarrow{\mathcal{E}}$ is a square dense matrix equal in dimension to the number of projected loci. Moreover, it will vary over replicates such that the complete covariance structure across all replicates is a large block-diagonal matrix with large blocks. Given the computational burden this entails we fitted approximate models to the simulated data where $\overrightarrow{\mathcal{E}}$ was diagonalised. For the same reason, we also used a diagonalised $\mathbf{Q}$ matrix which only contains information on coverage rather than complete information on the number of reads overlapping pairs of sites. In addition we also assumed $\mathcal{L}_{t_m,m} = \mathcal{L}_{\tau_m,m} = \mathbf{L}_0^{'}$ for all m. For real data we advocate not using these approximations.

Estimation error in allele frequency at a single-site is comparable to drift, with coverage O_{ii}, or effective coverage $\sigma_o^2 O_{ii}$, equivalent to N_E. Because of this it might be tempting to think that estimates of $V_{\bar{A}}(0)$ would remain unbiased if estimation error was ignored since the residual variance will soak up the excess variance when model fitting. The only advantage, then, of including $\overrightarrow{\mathcal{E}}$ is to increase precision when there is heterogeneity in coverage across sites. However, this is not the case - estimates of $V_{\bar{A}}(0)$ will be downwardly biased if estimation error is ignored and this bias can be substantial unless reads are very long and/or coverage is high. The reason for this is that a major source of information on $V_{\bar{A}}(0)$ comes from correlated changes in allele frequency at loci that are in LD, and in particular correlations that are tighter than expected under drift. If reads only span single sites then the error covariance in allele frequency is zero since $O_{ij} = 0$ resulting in correlations that are weaker than under drift which partly destroys the signal of selection. If reads are longer, then the error covariance in allele frequency will come to resemble that caused by drift and any bias when ignoring the errors should be reduced. However, at very long read lengths the bias may even become positive as the sampling correlations between distant sites on the same chromosome will be higher than that under drift where it will be broken down by a round of recombination (Equation S14).

S7. Comparison with the method of Buffalo and Coop (2019)

To make clearer the distinction between the approach of B&C and our approach, here we explicitly express the expectations and covariances appearing in B&C as conditional on $\mathbf{B}$ and $\boldsymbol{\alpha}$. In the sections dealing with our theory and inference, the conditioning (on $\mathbf{L}_0$) is left implicit. B&C work with the quantity

$$COV(\Delta\mathbf{p}_t, \Delta\mathbf{p}_\tau^\top | \mathbf{B}, \boldsymbol{\alpha}) \tag{S38}$$

where τ is some generation after t (B&C actually only work with the diagonal elements of this matrix, but we retain the full multi-locus model here for generality). Importantly, when B&C estimate the additive genetic variance in fitness they assume that both $\Delta\mathbf{p}_t$ and $\Delta\mathbf{p}_\tau$ both represent allele frequency change over a single generation (Assumption A). As the change due to drift will be independent in different generations, we can rewrite B&C's covariance:

$$\begin{aligned} COV(\Delta\mathbf{p}_t, \Delta\mathbf{p}_\tau^\top | \mathbf{B}, \boldsymbol{\alpha}) = & \; COV(\mathbf{L}_t\boldsymbol{\alpha}_t, \boldsymbol{\alpha}_\tau^\top \mathbf{L}_\tau | \mathbf{B}, \boldsymbol{\alpha}) \\ = & \; E\left[\mathbf{L}_t\boldsymbol{\alpha}_t\boldsymbol{\alpha}_\tau^\top \mathbf{L}_\tau | \mathbf{B}, \boldsymbol{\alpha}\right] - E\left[\mathbf{L}_t | \mathbf{B}, \boldsymbol{\alpha}\right] \boldsymbol{\alpha}_t\boldsymbol{\alpha}_\tau^\top E\left[\mathbf{L}_\tau | \mathbf{B}, \boldsymbol{\alpha}\right] \end{aligned} \tag{S39}$$

Since the diagonal elements of $\mathbf{L}$ have to be positive, a sufficient, but not necessary, condition for the final term to be zero is that there is no direct selection on the loci (i.e. $\boldsymbol{\alpha} = \mathbf{0}$). It is not a necessary condition because the change caused by direct selection at all loci could be exactly balanced by the change caused by indirect selection at other loci, although we ignore this unlikely scenario. The assumption that $\boldsymbol{\alpha} = \mathbf{0}$ is achieved in B&C by assuming that sites can be partitioned into neutral and selected sites and that allele frequency change is only tracked at the neutral sites (Assumption B). To understand the consequences of this assumption we consider all loci are being followed, both a selected set ($\mathcal{S}$) and a neutral set ($\mathcal{N}$). Consequently,

$$\boldsymbol{\alpha} = \begin{bmatrix} \mathbf{0} \\ \boldsymbol{\alpha}_{\mathcal{S}} \end{bmatrix} \tag{S40}$$

and so

$$\boldsymbol{\alpha}\boldsymbol{\alpha}^\top = \begin{bmatrix} \mathbf{0} & \mathbf{0} \\ \mathbf{0} & \boldsymbol{\alpha}_{\mathcal{S}}\boldsymbol{\alpha}_{\mathcal{S}}^\top \end{bmatrix} \tag{S41}$$

We can also partition $\mathbf{L}$

$$\mathbf{L} = \begin{bmatrix} \mathbf{L}_{\mathcal{N}} & \mathbf{L}_{\mathcal{N},\mathcal{S}} \\ \mathbf{L}_{\mathcal{S},\mathcal{N}} & \mathbf{L}_{\mathcal{S}} \end{bmatrix} \tag{S42}$$

and writing $\mathbf{L}_{\mathcal{N},\mathcal{S}} = \mathbf{B}_{\mathcal{N}}\mathbf{R}_{\mathcal{N},\mathcal{S}}\mathbf{B}_{\mathcal{S}}$, Equation S39 for neutral sites becomes

$$\begin{aligned} COV(\Delta\mathbf{p}_{\mathcal{N}_t}, \Delta\mathbf{p}_{\mathcal{N}_\tau}^\top | \mathbf{B}, \boldsymbol{\alpha}) = & \; \mathbf{B}_{\mathcal{N}_t} E\left[\mathbf{R}_{\mathcal{N}_t,\mathcal{S}_t}\mathbf{B}_{\mathcal{S}_t}\boldsymbol{\alpha}_{\mathcal{S}_t}\boldsymbol{\alpha}_{\mathcal{S}_\tau}^\top \mathbf{B}_{\mathcal{S}_\tau}\mathbf{R}_{\mathcal{S}_\tau,\mathcal{N}_\tau} | \mathbf{B}, \boldsymbol{\alpha}\right] \mathbf{B}_{\mathcal{N}_\tau} \\ & + \mathbf{B}_{\mathcal{N}_t} E\left[\mathbf{R}_{\mathcal{N}_t,\mathcal{S}_t} | \mathbf{B}, \boldsymbol{\alpha}\right] \mathbf{B}_{\mathcal{S}_t}\boldsymbol{\alpha}_{\mathcal{S}_t}\boldsymbol{\alpha}_{\mathcal{S}_\tau}^\top \mathbf{B}_{\mathcal{S}_\tau} E\left[\mathbf{R}_{\mathcal{S}_\tau,\mathcal{N}_\tau} | \mathbf{B}, \boldsymbol{\alpha}\right] \mathbf{B}_{\mathcal{N}_\tau} \end{aligned} \tag{S43}$$

Under the conditioning of B&C, $\mathbf{R}_{\mathcal{S},\mathcal{N}_\tau}$ is a random variable. If we assume that the linkage-disequilibrium between the neutral alleles and the selected alleles has arbitrary sign, then $E[\mathbf{R}_{\mathcal{S},\mathcal{N}_\tau} | \mathbf{B}, \boldsymbol{\alpha}] = \mathbf{0}$, which will be met if the reference allele is chosen arbitrarily

(e.g. not based on minor allele frequency (Good 2022)). Under this assumption (Assumption C) the final term in Equation S43 disappears to give:

$$COV(\Delta \mathbf{p}_{\mathcal{N}_t}, \Delta \mathbf{p}_{\mathcal{N}_\tau}^\top | \mathbf{B}, \boldsymbol{\alpha}) = \mathbf{B}_{\mathcal{N}_t} E\left[\mathbf{R}_{\mathcal{N}_t,\mathcal{S}_t} \mathbf{B}_{\mathcal{S}_t} \boldsymbol{\alpha}_{\mathcal{S}_t} \boldsymbol{\alpha}_{\mathcal{S}_\tau}^\top \mathbf{B}_{\mathcal{S}_\tau} \mathbf{R}_{\mathcal{S}_\tau,\mathcal{N}_\tau} | \mathbf{B}, \boldsymbol{\alpha}\right] \mathbf{B}_{\mathcal{N}_\tau} \tag{S44}$$

As in our inference section, the vector of allele frequency changes could be transformed using matrix $\mathbf{P}$ ($\Delta \overrightarrow{\mathbf{p}} = \mathbf{P}\Delta\mathbf{p}$) and B&C use the projection $\mathbf{P} = \mathbf{B}_{\mathcal{N}_t}^{-1}$ (they actually multiply this by $\sqrt{2}$ - see Equation S54) which results in

$$\begin{aligned} COV(\Delta \overrightarrow{\mathbf{p}}_{\mathcal{N}_t}, \Delta \overrightarrow{\mathbf{p}}_{\mathcal{N}_\tau}^\top | \mathbf{B}, \boldsymbol{\alpha}) &= E\left[\mathbf{R}_{\mathcal{N}_t,\mathcal{S}_t} \mathbf{B}_{\mathcal{S}_t} \boldsymbol{\alpha}_{\mathcal{S}_t} \boldsymbol{\alpha}_{\mathcal{S}_\tau}^\top \mathbf{B}_{\mathcal{S}_\tau} \mathbf{R}_{\mathcal{S}_\tau,\mathcal{N}_\tau} | \mathbf{B}, \boldsymbol{\alpha}\right] \mathbf{B}_{\mathcal{N}_\tau} \mathbf{B}_{\mathcal{N}_t}^{-1} \\ &= \mathbf{B}_{\mathcal{N}_t}^{-1} E\left[\mathbf{R}_{\mathcal{N}_t,\mathcal{S}_t} \mathbf{B}_{\mathcal{S}_t} \boldsymbol{\alpha}_{\mathcal{S}_t} \boldsymbol{\alpha}_{\mathcal{S}_\tau}^\top \mathbf{B}_{\mathcal{S}_\tau} \mathbf{R}_{\mathcal{S}_\tau,\mathcal{N}_\tau} | \mathbf{B}, \boldsymbol{\alpha}\right] \mathbf{B}_{\mathcal{N}_\tau} \end{aligned} \tag{S45}$$

Further derivation in B&C considers the expected value of a diagonal element of this matrix: the average (over neutral loci) covariance in projected allele frequency change. However, it is perhaps easier to note that the trace of this matrix is equal to this average multiplied by the number of neutral loci, $n_{L_\mathcal{N}}$ (see below). Since the trace of an outer product is equal to the inner product we get:

$$\begin{aligned} Tr\left(COV(\Delta \overrightarrow{\mathbf{p}}_{\mathcal{N}_t}, \Delta \overrightarrow{\mathbf{p}}_{\mathcal{N}_\tau}^\top | \mathbf{B}, \boldsymbol{\alpha})\right) &= E\left[\boldsymbol{\alpha}_{\mathcal{S}_t}^\top \mathbf{B}_{\mathcal{S}_t} \mathbf{R}_{\mathcal{S}_t,\mathcal{N}_t} \mathbf{B}_{\mathcal{N}_t}^{-1} \mathbf{B}_{\mathcal{N}_\tau} \mathbf{R}_{\mathcal{N}_\tau,\mathcal{S}_\tau} \mathbf{B}_{\mathcal{S}_\tau} \boldsymbol{\alpha}_{\mathcal{S}_\tau} | \mathbf{B}, \boldsymbol{\alpha}\right] \\ &= \boldsymbol{\alpha}_{\mathcal{S}_\tau}^\top \mathbf{B}_{\mathcal{S}_t} E\left[\mathbf{R}_{\mathcal{S}_t,\mathcal{N}_t} \mathbf{B}_{\mathcal{N}_t}^{-1} \mathbf{B}_{\mathcal{N}_\tau} \mathbf{R}_{\mathcal{N}_\tau,\mathcal{S}_\tau} | \mathbf{B}, \boldsymbol{\alpha}\right] \mathbf{B}_{\mathcal{S}_\tau} \boldsymbol{\alpha}_{\mathcal{S}_\tau} \end{aligned} \tag{S46}$$

The diagonal element j of $\mathbf{W}_{t\tau} = \mathbf{R}_{\mathcal{S}_t,\mathcal{N}_t} \mathbf{B}_{\mathcal{N}_t}^{-1} \mathbf{B}_{\mathcal{N}_\tau} \mathbf{R}_{\mathcal{N}_\tau,\mathcal{S}_\tau}$ is equal to the sum of selected locus j's $R_{t,ji} R_{\tau,ji} (b_{\tau,i}/b_{t,i})$ across all neutral loci i. The jk^{th} off-diagonal element is the sum of $R_{t,ji} R_{\tau,ki} (b_{\tau,i}/b_{t,i})$ for selected loci j and k across all neutral loci i. If we write $\mathbf{W}_{t\tau} = \mathbf{H}_{t\tau} + (\mathbf{W}_{t\tau} - \mathbf{H}_{t\tau})$ where $\mathbf{H}_{t\tau}$ and $\mathbf{W}_{t\tau} - \mathbf{H}_{t\tau}$ are zero but for the diagonal and off-diagonal elements respectively, then Equation S46 becomes

$$\begin{aligned} Tr\left(COV(\Delta \overrightarrow{\mathbf{p}}_{\mathcal{N}_t}, \Delta \overrightarrow{\mathbf{p}}_{\mathcal{N}_\tau}^\top | \mathbf{B}, \boldsymbol{\alpha})\right) &= \boldsymbol{\alpha}_{\mathcal{S}_t}^\top \mathbf{B}_{\mathcal{S}_t} E\left[\mathbf{H}_{t\tau} | \mathbf{B}, \boldsymbol{\alpha}\right] \mathbf{B}_{\mathcal{S}_\tau} \boldsymbol{\alpha}_{\mathcal{S}_\tau} \\ &\quad + \boldsymbol{\alpha}_{\mathcal{S}_t}^\top \mathbf{B}_{\mathcal{S}_t} E\left[\mathbf{W}_{t\tau} - \mathbf{H}_{t\tau} | \mathbf{B}, \boldsymbol{\alpha}\right] \mathbf{B}_{\mathcal{S}_\tau} \boldsymbol{\alpha}_{\mathcal{S}_\tau} \end{aligned} \tag{S47}$$

If we focus on a system with two selected loci, j and k, then the final term in Equation S47 is equal to:

$$\sum_j \sum_k \left(\alpha_{t,j} \alpha_{\tau,k} b_{j_t,j_t} b_{k_\tau,k_\tau} \sum_i \left(E\left[R_{t,ij} R_{\tau,ik} | \mathbf{B}, \boldsymbol{\alpha}\right] b_{i_\tau}/b_{i_t} \right) + \alpha_{j_\tau} \alpha_{k_t} b_{j_\tau,j_\tau} b_{k_t,k_t} \sum_i \left(E\left[R_{\tau,ij} R_{t,ik} | \mathbf{B}, \boldsymbol{\alpha}\right] b_{i_\tau}/b_{i_t} \right) \right). \tag{S48}$$

Under Hill-Robertson interference, if α_j and α_k have the same sign we expect them to be in negative LD with each other, and as a consequence have opposing patterns of LD with the neutral loci (i.e. if $\alpha_j \alpha_k > 0$ then we expect $E[R_{i,j} R_{i,k}] < 0$ and vice versa). Although the terms of these products are evaluated at different generations in Equation S48 (t and τ) we expect the terms to share sign in the same way, generating a negative expectation for the second term in Equation S47. However, assuming an absence of Hill-Robertson interference, or signed linkage-disequilibrium more generally (Assumption D), then the final term in Equation S47 can be dropped:

$$Tr\left(COV(\Delta \overrightarrow{\mathbf{p}}_{\mathcal{N}_t}, \Delta \overrightarrow{\mathbf{p}}_{\mathcal{N}_\tau}^\top) | \mathbf{B}, \boldsymbol{\alpha}\right) = \boldsymbol{\alpha}_{\mathcal{S}_t}^\top \mathbf{B}_{\mathcal{S}_t} E\left[\mathbf{H}_{t\tau} | \mathbf{B}, \boldsymbol{\alpha}\right] \mathbf{B}_{\mathcal{S}_\tau} \boldsymbol{\alpha}_{\mathcal{S}_\tau} \tag{S49}$$

Based on a deterministic model for changes in linkage-disequilibrium (Assumption E) and assuming nongametic-phase linkage-disequilibrium is absent (Assumption F), Equations 40-44 in B&C derive an expression from which $E\left[\mathbf{H}_{t\tau} | \mathbf{B}, \boldsymbol{\alpha}\right]$ can be computed. Under the assumption that drift (or selection) does not alter the dynamics of $\mathbf{R}$ then (Equation 42 in B&C):

$$L_{j_\tau,i_\tau} = L_{j_t,i_t} \frac{b_{j_\tau}^2}{b_{j_t}^2} (1 - r(g_{j,i}))^{\tau - t} \tag{S50}$$

which implies

$$R_{j_\tau,i_\tau} = R_{j_t,i_t} \frac{b_{j_\tau} b_{i_t}}{b_{j_t} b_{i_\tau}} (1 - r(g_{j,i}))^{\tau - t} \tag{S51}$$

and so

$$\begin{aligned} E\left[H_{j_t,j_\tau} | \mathbf{B}, \boldsymbol{\alpha}\right] &= \textstyle\sum_i E\left[R_{j_t,i_t} R_{j_\tau,i_\tau} | \mathbf{B}, \boldsymbol{\alpha}\right] \frac{b_{i_\tau}}{b_{i_t}} \\ &= \frac{b_{j_\tau}}{b_{j_t}} \textstyle\sum_i E\left[R_{j_t,i_t}^2 | \mathbf{B}, \boldsymbol{\alpha}\right] (1 - r(g_{j,i}))^{\tau - t} \end{aligned} \tag{S52}$$

where $r(g_{j,i})$ is the recombination rate as a function of the distance $g_{i,j}$ between the two loci. Writing $\mathbf{F}_{t\tau}$ a diagonal matrix with the jth element equal to $\sum_i E\left[R_{j_t,i_t}^2 | \mathbf{B}, \boldsymbol{\alpha}\right] (1 - r(g_{j,i}))^{\tau - t}$, then $E\left[\mathbf{H}_{t\tau} | \mathbf{B}, \boldsymbol{\alpha}\right] = E\left[\mathbf{F}_{t\tau} | \mathbf{B}, \boldsymbol{\alpha}\right] \mathbf{B}_{\mathcal{S}_\tau} \mathbf{B}_{\mathcal{S}_t}^{-1}$ and so

$$
\begin{aligned}
Tr\left(COV(\Delta\overrightarrow{\mathbf{p}}_{\mathcal{N}_t},\Delta\overrightarrow{\mathbf{p}}^{\top}_{\mathcal{N}_\tau}|\mathbf{B},\boldsymbol{\alpha})\right) &= \boldsymbol{\alpha}^{\top}_{\mathcal{S}_t}\mathbf{B}_{\mathcal{S}_t}E\left[\mathbf{F}_{t\tau}|\mathbf{B},\boldsymbol{\alpha}\right]\mathbf{B}_{\mathcal{S}_\tau}\mathbf{B}^{-1}_{\mathcal{S}_t}\mathbf{B}_{\mathcal{S}_\tau}\boldsymbol{\alpha}_{\mathcal{S}_\tau} \\
&= \boldsymbol{\alpha}^{\top}_{\mathcal{S}_t}E\left[\mathbf{F}_{t\tau}|\mathbf{B},\boldsymbol{\alpha}\right]\mathbf{B}_{\mathcal{S}_\tau}\mathbf{B}_{\mathcal{S}_\tau}\boldsymbol{\alpha}_{\mathcal{S}_\tau} \\
&= \boldsymbol{\alpha}^{\top}_{\mathcal{S}_t}E\left[\mathbf{F}_{t\tau}|\mathbf{B},\boldsymbol{\alpha}\right]\mathbf{B}^{2}_{\mathcal{S}_\tau}\boldsymbol{\alpha}_{\mathcal{S}_\tau}
\end{aligned}
\tag{S53}
$$

where (under random mating) $\mathbf{B}^2_{\mathcal{S}_\tau}$ is a diagonal matrix with elements proportional to the genetic diversities in generation τ. However, this is still hard to evaluate since $E\left[\mathbf{F}_{t\tau}|\mathbf{B},\boldsymbol{\alpha}\right]$ will vary over loci in a way that may depend on $\mathbf{B}$ and hence $\mathbf{B}^2_{\mathcal{S}_t}$. The method of B&C assumes that the sample correlation between the diagonal elements of $E\left[\mathbf{F}_{t\tau}|\mathbf{B},\boldsymbol{\alpha}\right]$ and the elements $\alpha_{j_t}\alpha_{j_\tau}b^2_{j_\tau}$ is zero (Assumption G). Under this assumption:

$$
\begin{aligned}
Tr\left(COV(\Delta\overrightarrow{\mathbf{p}}_{\mathcal{N}_t},\Delta\overrightarrow{\mathbf{p}}^{\top}_{\mathcal{N}_\tau}|\mathbf{B},\boldsymbol{\alpha})\right) &= \frac{1}{n_{L_\mathcal{S}}}\boldsymbol{\alpha}^{\top}_{\mathcal{S}_t}\mathbf{B}^{2}_{\mathcal{S}_\tau}\boldsymbol{\alpha}_{\mathcal{S}_\tau}E\left[Tr(\mathbf{F}_{t\tau})|\mathbf{B},\boldsymbol{\alpha}\right] \\
&= \frac{1}{n_{L_\mathcal{S}}}C_a(t\rightarrow\tau)E\left[Tr(\mathbf{F}_{t\tau})|\mathbf{B},\boldsymbol{\alpha}\right]
\end{aligned}
\tag{S54}
$$

where $n_{L_\mathcal{S}}$ is the number of selected loci (note that in B&C the leading term is $\frac{1}{2n_{L_\mathcal{S}}}$ not $\frac{1}{n_{L_\mathcal{S}}}$ and this is because our projection matrices are only proportional by a factor $\sqrt{2}$ - see Equation S45). The method of B&C further assumes (Assumption H) that the average effects are constant in time such that $\boldsymbol{\alpha}_t = \boldsymbol{\alpha}_\tau$ then this reduces to

$$
Tr\left(COV(\Delta\overrightarrow{\mathbf{p}}_{\mathcal{N}_t},\Delta\overrightarrow{\mathbf{p}}^{\top}_{\mathcal{N}_\tau}|\mathbf{B},\boldsymbol{\alpha})\right) = \frac{V_a(\tau)}{n_{L_\mathcal{S}}}E\left[Tr(\mathbf{F}_{t\tau})|\mathbf{B},\boldsymbol{\alpha}\right]
\tag{S55}
$$

Under Assumption H, Assumption G implies that the additive genic variance contributed by a selected locus is independent of its associations with neutral loci as measured by $R^2_{i_t,j_t}$. However, allele frequencies at selected loci enter the expression for the additive genic variance, and they also dictate the range of R_{i_t,j_t} and therefore the magnitude of $R^2_{i_t,j_t}$: when selected allele frequencies are small compared to frequencies at neutral alleles, R_{i_t,j_t} cannot cover the full range of -1 to 1 (Sved and Hill 2018).

B&C approximate $\mathbf{R}_t$, and therefore $\mathbf{F}_{t\tau}$ under the assumption of mutation-drift-recombination equilibrium (Assumption I). Ohta and Kimura (1971) derived the expectation of $L_{j_t,i_t}L_{j_t,i_t} = L^2_{j_t,i_t}$ under this assumption, although the expectation of $R^2_{j_t,i_t}$ can only be approximated as the expectation of $L^2_{j_t,i_t}$ divided by the expectation of the genetic diversities at the two loci, and is only accurate when the minor allele frequencies are greater than 10% (McVean 2002) – and can be out by orders of magnitude when allele frequencies are extreme (Song and Song 2007), as can be expected at loci under selection. Moreover, Ohta and Kimura (1971) derived the expectation $E[L^2_{j_t,i_t}]$ yet the B&C approach actually requires $E\left[L^2_{j_t,i_t}|\mathbf{B},\boldsymbol{\alpha}\right]$ which is considerably more challenging to compute (Good 2022). In the Appendix B&C also relax, to some extent, Assumption I, where $Tr(\mathbf{F}_{t\tau})$ is calculated empirically using all loci, neutral and selected (Equation 55). In both cases - using the neutral expectation (Assumption I) or empirical LD (Assumption I-b) - the expected LD between selected and neutral loci will be overestimated since in reality selected alleles will be rarer than neutral alleles and so their LD, even measured as a correlation, will be reduced compared to that between neutral loci (Sved and Hill 2018).

Equation S55 allows $V_a(\tau)$ to be estimated, but B&C aim to estimate $V_a(t)$. Under Assumption H, and assuming that the proportional change in genetic diversity at selected loci between generation t and τ is constant across loci (Assumption J: $\mathbf{B}^2_{\mathcal{S}_t}\mathbf{B}^{-2}_{\mathcal{S}_\tau} = \phi_{t,\tau}\mathbf{I}$), $V_a(t) = \phi_{t,\tau}V_a(\tau)$.

The above derivation assumes that the sites can be partitioned into selected and neutral sites and that the map positions of all sites are known. In practice, this will often be infeasible and so a number of additional assumptions are required. In the absence of map positions, Haldane's (1919) mapping function is assumed for $r(g)$ (Assumption K) but, since the selected loci and their physical position, g, are assumed unknown, a model is also required for g. B&C assume that selected and neutral loci are distributed uniformly and independently such that $g_{j,i}$ has a triangular distribution (Assumption L). Also, since the selected sites are unobserved, $\phi_{t,\tau}$ cannot be computed and so it is assumed that that $\phi_{t,\tau}$ is equal to the ratio of genetic diversity at generation τ to genetic diversity at generation t across all neutral loci (Assumption M).

In addition to the assumptions/approximations made when developing the theory, additional assumptions/approximations are made when making inferences from data. Rather than taking the average (over loci) covariance (over evolutionary replicates) the covariance over loci is taken. However, from the law of total covariance this is expected to yield the correct result under Assumptions B and C. In addition, rather than calculating the (co)variances in projected allele frequency change, it is assumed (Assumption N) that the (co)variances in actual allele frequency divided through by the average projection is a good approximation: $COV_L(\Delta p_t, \Delta p_\tau)/E[b^2_t]$ is a good approximation of $COV_L(\Delta p_t/b_t, \Delta p_\tau/b_t)$ where COV_L designates a covariance over loci (Equation 16 B&C). The two quantities are only expected to agree under restrictive conditions (Bohrnstedt and Goldberger 1969). However, it is not necessary to make Assumption N and relaxing it can reduce the bias in the estimator considerably. Finally, if there is measurement error in allele frequencies and the same allele frequency measurements are used to calculate change over adjacent intervals then this will generate downward bias in the estimated covariances. This is corrected for in B&C by assuming the sampling error in allele frequencies is binomial around the true value (Assumption O), although in practice non-binomial causes of overdispersion are likely.

To connect Equation S55 with the derivation in B&C more clearly, we can write

$$Tr\left(COV(\Delta\overrightarrow{\mathbf{p}}_{\mathcal{N}_t},\Delta\overrightarrow{\mathbf{p}}^{\top}_{\mathcal{N}_\tau}|\mathbf{B},\boldsymbol{\alpha})\right) = n_{L_\mathcal{N}}\overline{COV(\Delta\overrightarrow{p}_{\mathcal{N}_t},\Delta\overrightarrow{p}^{\top}_{\mathcal{N}_\tau}|\mathbf{B},\boldsymbol{\alpha})} \tag{S56}$$

where the overline denotes the sample mean for the $n_{L_\mathcal{N}}$ neutral loci. Similarly,

$$\begin{aligned} Tr(\mathbf{F}_{t\tau}) &= \sum_j\sum_i E\left[R^2_{jt,i_t}|\mathbf{B},\boldsymbol{\alpha}\right](1-r(g_{j,i}))^{\tau-t} \\ &= n_{L_\mathcal{N}}\overline{\sum_j E\left[R^2_{jt,\mathcal{N}_t}|\mathbf{B},\boldsymbol{\alpha}\right](1-r(g_{j,\mathcal{N}}))^{\tau-t}} \end{aligned} \tag{S57}$$

Equation S55 can then be written as

$$\overline{COV(\Delta\overrightarrow{p}_{\mathcal{N}_t},\Delta\overrightarrow{p}^{\top}_{\mathcal{N}_\tau}|\mathbf{B},\boldsymbol{\alpha})} = \frac{V_a(\tau)}{n_{L_\mathcal{S}}}\overline{\sum_j E\left[R^2_{jt,\mathcal{N}_t}|\mathbf{B},\boldsymbol{\alpha}\right](1-r(g_{j,\mathcal{N}}))^{\tau-t}} \tag{S58}$$

which is Equation 8 in B&C averaged over neutral loci and can be further simplified to

$$\overline{COV(\Delta\overrightarrow{p}_{\mathcal{N}_t},\Delta\overrightarrow{p}^{\top}_{\mathcal{N}_\tau}|\mathbf{B},\boldsymbol{\alpha})} = V_a(\tau)\overline{E\left[R^2_{\mathcal{S}_t,\mathcal{N}_t}|\mathbf{B},\boldsymbol{\alpha}\right](1-r(g_{\mathcal{S},\mathcal{N}}))^{\tau-t}} \tag{S59}$$

where the overline indicates the sample average of all the $n_{L_\mathcal{S}}n_{L_\mathcal{N}}$ pairwise comparisons between selected loci and neutral loci.

Extending this theory to allele frequency change measured in independent replicates, rather than at different time-points in a single population is straightforward (Buffalo and Coop 2020). If allele frequency change is measured in two populations, m and n, both of which have been derived independently from the base population t generation in the past, Equation S59 can be expressed as:

$$\overline{COV(\Delta\overrightarrow{p}_{\mathcal{N}_m},\Delta\overrightarrow{p}^{\top}_{\mathcal{N}_n}|\mathbf{B},\boldsymbol{\alpha})} = V_a(m,n)\overline{E\left[R^2_{\mathcal{S}_0,\mathcal{N}_0}|\mathbf{B},\boldsymbol{\alpha}\right](1-r(g_{\mathcal{S},\mathcal{N}}))^{2t}} \tag{S60}$$

assuming the average effects have stayed constant.

S8. N_E and average effects under a log-linear model

In our simulations, we model the logarithm of absolute fitness of an individual k using an additive model across loci:

$$log(W_k) = Y_k = \sum_{i=1}^{n_L} y_{k,i} + e_k$$

where $y_{k,i}$ is the genotypic contribution of locus i to the log absolute fitness of individual k, and the environmental deviations, e, have zero mean and standard deviation σ_e. We allow within-locus deviations from additivity using a fitness-scheme equivalent to the one employed by Lynch and Walsh (1998) (pp. 67) adapted for *proportions* of reference alleles as opposed to counts:

$$y_{k,i} = \begin{cases} 0 & \text{if } c_{k,i} = 0 \\ (1+\kappa_i)\eta_i/2 & \text{if } c_{k,i} = 0.5 \\ \eta_i & \text{if } c_{k,i} = 1 \end{cases} \tag{S61}$$

Note that in the parameterisation of Falconer and Mackay (1996), the genotypic value of the heterozygotes is $(\eta_i + d_i)/2$ such that $d_i = \kappa_i\eta_i$.

We then draw $2N_{t+1}$ parents with replacement from a multinomial with the probability of k being a parent of an offspring proportional to its absolute fitness $W_k = exp(Y_k)$. Following Kojima (1959) we can define Fisher's average effect for relative fitness in terms of the partial derivative of mean fitness with respect to each $E[c] = p_0$ and then rescale by mean fitness:

$$\begin{aligned} \alpha_i &= \frac{1}{\bar{W}}\frac{\partial\bar{W}}{\partial p_{0_i}} \\ &= \frac{1}{E[exp(Y)]}\frac{\partial E[exp(Y)]}{\partial p_{0_i}} \\ &= \frac{\partial log(E[exp(Y)])}{\partial p_{0_i}} \end{aligned} \tag{S62}$$

Assuming Y to be normally distributed with mean μ_Y and variance V_Y allows us to use the expression for the expectation of a log-normal distribution to write:

$$\begin{aligned} \alpha_i &= \frac{\partial log(exp(\mu_Y + V_Y/2))}{\partial p_i} \\ &= \frac{\partial(\mu_Y + V_Y/2)}{\partial p_i} \end{aligned} \tag{S63}$$

It is worth highlighting that, so far, we have made no assumptions about the distribution of the contributions (y_i) made by individual loci to Y.

Next, we assume Hardy-Weinberg genotypic frequencies to write:

$$\begin{aligned}\mu_Y &= \textstyle\sum_{i=1}^{n_L} E[y_i] \\ &= \textstyle\sum_{i=1}^{n_L} [p_i^2\eta_i + 2p_iq_i(1+\kappa_i)\eta_i/2] \\ &= \textstyle\sum_{i=1}^{n_L} [p_i\eta_i + p_iq_i\kappa_i\eta_i] \\ &= \textstyle\sum_{i=1}^{n_L} \eta_i[p_i + p_iq_i\kappa_i]\end{aligned} \tag{S64}$$

Differentiating with respect to p_i yields:

$$\frac{\partial\mu_y}{\partial p_i} = \eta_i[1+\kappa_i(q_i-p_i)] \tag{S65}$$

Assuming that all additive genetic variance is genic:

$$\begin{aligned}V_Y &= \textstyle\sum_{i=1}^{n_L} VAR(y_i) + \sigma_e^2 \\ &= \textstyle\sum_{i=1}^{n_L} (E[y_i^2] - E[y_i]^2) + \sigma_e^2 \\ &= \textstyle\sum_{i=1}^{n_L} [p_i^2\eta_i^2 + 2p_iq_i(1+\kappa_i)^2\eta_i^2/4 - \eta_i^2(p_i + p_iq_i\kappa_i)^2] + \sigma_e^2 \\ &= \textstyle\sum_{i=1}^{n_L} [p_iq_i(1+\kappa_i)^2\eta_i^2/2 - \eta_i^2(2p_i^2q_i\kappa_i + p_i^2q_i^2\kappa_i^2)] + \sigma_e^2 \\ &= \textstyle\sum_{i=1}^{n_L} [\frac{p_iq_i}{2}(1+\kappa_i)^2\eta_i^2 - \eta_i^2p_i^2q_i\kappa_i(2+q_i\kappa_i)] + \sigma_e^2\end{aligned} \tag{S66}$$

Differentiating with respect to p_i:

$$\begin{aligned}\frac{\partial V_y}{\partial p_i} &= \frac{q_i-p_i}{2}(1+\kappa_i)^2\eta_i^2 - \eta_i^2\kappa_i[(2p_iq_i - p_i^2)(2+\kappa_iq_i) - p_i^2q_i\kappa_i] \\ &= \frac{q_i-p_i}{2}(1+\kappa_i)^2\eta_i^2 - p_i\eta_i^2\kappa_i[(2q_i - p_i)(2+\kappa_iq_i) - p_iq_i\kappa_i]\end{aligned} \tag{S67}$$

Substituting expressions for $\frac{\partial\mu_y}{\partial p_i}$ and $\frac{\partial V_y}{\partial p_i}$ yields:

$$\begin{aligned}\alpha_i &= \eta_i[1+\kappa_i(q_i-p_i)] + \\ &\quad \frac{q_i-p_i}{4}(1+\kappa_i)^2\eta_i^2 - \frac{p_i\eta_i^2\kappa_i}{2}[(2q_i-p_i)(2+\kappa_iq_i) - p_iq_i\kappa_i]\end{aligned} \tag{S68}$$

In the parameterisation of Falconer and Mackay (1996), this translates to:

$$\begin{aligned}\alpha_i &= \eta_i + d_i(q_i-p_i) + \\ &\quad \frac{q_i-p_i}{4}(\eta_i+d_i)^2 - \frac{d_ip_i}{2}[(2q_i-p_i)(2\eta_i+q_id_i) - p_iq_id_i]\end{aligned} \tag{S69}$$

Note that as a first approximation Equations S69 and S68 reduce to the classical quantitative genetic expressions for the average effect of gene substitution (Equation 7.5 in Falconer and Mackay (1996) and Equation 4.10b in Lynch and Walsh (1998)).

In order to calculate N_E under this multinomial log-linear model we need to know the variance in offspring number V_o caused by any environmental or non-additive genetic variance. Then $N_E = 4N/(2+V_o)$ where N is the census population size (Wright 1938). The expected number of offspring for parent k is $2N_{t+1}p_k$, as given above, and the variance in the number offspring is $2N_{t+1}p_k(1-p_k)$. From the the law of total variance $V_o = 4N_{t+1}^2Var(p) + 2N_{t+1}E[p(1-p)]$. Since $E[p(1-p)] = E[p] - E[p^2]$ and $Var(p) = E[p^2] - E[p]^2$ then $E[p(1-p)] = E[p] - Var(p) + E[p]^2$. Since $E[p] = 1/N_t$ by definition,

$$\begin{aligned}V_o = &\ 4N_{t+1}^2Var(p) + 2N_{t+1}E[p(1-p)] \\ = &\ 4N_{t+1}^2Var(p) + \frac{2N_{t+1}}{N_t} - 2N_{t+1}Var(p) + \frac{2N_{t+1}}{N_t^2} \\ = &\ (4N_{t+1}^2 - 2N_{t+1})Var(p) + \frac{2N_{t+1}}{N_t} + \frac{2N_{t+1}}{N_t^2}\end{aligned} \tag{S70}$$

From the properties of the log-normal (with zero mean) we know $E[P] = exp(\sigma_e^2/2)$ and $Var(P) = (exp(\sigma_e^2) - 1)exp(\sigma_e^2)$. Since $p = P/(N_tE[P])$

$$\begin{aligned}Var(p) = &\ Var(P)/(N_tE[P])^2 \\ = &\ (exp(\sigma_e^2) - 1)exp(\sigma_e^2)/(N_texp((\sigma_e^2)/2))^2 \\ = &\ (exp(\sigma_e^2) - 1)exp(\sigma_e^2)/(N_t^2exp(\sigma_e^2)) \\ = &\ (exp(\sigma_e^2) - 1)/N_t^2\end{aligned} \tag{S71}$$

If N_{t+1} and N_t are large then Equation S70 simplifies to

$$V_o = 4N_{t+1}^2Var(p) + 2N_{t+1}/N_t \tag{S72}$$

such that

$$V_o = (4N_{t+1}^2/N_t^2)(exp(\sigma_e^2) - 1) + 2N_{t+1}/N_t \tag{S73}$$

If the population size is constant then this simplifies to $V_o = 4exp(\sigma_e^2) - 2$ and the variance effective population size, N_{E_t}, is $4N_t/(2 + V_o) = N_t/exp(\sigma_e^2)$. Note however, that in these expressions using σ_e^2 only assumes the non-additive genetic variance is zero. When non-additive genetic variance is present (which there will be to a small degree even with additivity on the log-scale) V_o will be greater than predicted here and N_E will be consequently smaller.

S9. Workflow for implementing the method

Box 1. General workflow of the method

We use genome-wide allele frequency change data ($\Delta\mathbf{p}$) from independent evolutionary replicates derived from the same ancestral base population with a known linkage structure ($\mathbf{L}_0$) to infer the mean ($\boldsymbol{\mu}_{\tilde{\alpha}}$) and the variance ($\mathbf{V}_{\tilde{\alpha}}$) of the distribution of average effects for fitness. Our goal is to then estimate an expectation for the additive genetic variance for relative fitness by averaging over the distribution of average effects; i.e. $E[V_{\bar{A}}(0)] = Tr(\mathbf{L}_0\mathbf{V}_{\tilde{\alpha}}) + \boldsymbol{\mu}_{\tilde{\alpha}}^\top \mathbf{L}_0 \boldsymbol{\mu}_{\tilde{\alpha}}$. We use simple, yet biologically sensible models, $\boldsymbol{\mu}_{\tilde{\alpha}} = \beta_{\tilde{\alpha}}^{(1)}(\mathbf{p}_0 - \mathbf{q}_0)$ and $\mathbf{V}_{\tilde{\alpha}} = \sigma_{\tilde{\alpha}}^2 \mathbf{L}_0^{p_{\tilde{\alpha}}}$, which essentially allows the average effect for fitness at a site to depend on allele frequency and genetic diversity. This model can be readily implemented as a linear mixed model for suitably projected allele frequency change treating locus effects as random (see below). This, effectively, allows us to partition the total allele frequency change into a component caused by predictable responses to direct and indirect selection (modelled using the random effects of locus) and a component caused by unpredictable responses to selection as well as genetic drift (absorbed by the model residuals).

Experimental design and data:

A typical experimental design permitting the implementation of our method would involve a base populations of N_0 individuals which are sequenced individually. A number of independent evolutionary replicate populations derived from the base populations are then allowed to evolve between generations t and τ after the base population. If the replicate populations are derived from the base population after just a single round of reproduction t would be 1.

The following data are compiled from the experiment:

$\mathbf{C}_{\mathbf{g},\mathbf{0}}$: A matrix with $2N_0$ rows and n_L columns indicating the presence or absence (1 or 0) at each of the n_L segregating sites in each of the $2N_0$ gametic contributions in the base population. If phased genomes are unavailable, the $N_0 \times n_L$ matrix of the proportions of the copies of the (arbitrarily chosen) reference allele at each locus in each individual ($\mathbf{C}_\mathbf{0}$) may be used.

$\Delta\mathbf{p}_m$: For each replicate m, a vector representing allele frequency change at each segregating locus between generations t and τ after the base populations. t and τ must be known.

$\mathbf{R}_{-}$: An $n_L \times n_L$ matrix with 1's on the diagonal and the probabilities of non-recombination between pairs of sites as off-diagonal elements. A recombination map, if available, may be used to construct this matrix.

Step 1: Compute $\mathbf{L}_0$, $\mathbf{L}_0^{'}$, $\mathbf{L}_0^{''}$, and $\tilde{\mathbf{L}}_0$

First, we compute the covariance of $\mathbf{C}_\mathbf{0}$ over individuals to obtain $\mathbf{L}_0$, and the covariance of $\mathbf{C}_{\mathbf{g},\mathbf{0}}$ over gametic contributions to obtain the matrix of gametic phase disequilibria ($\mathbf{L}_0^{'}$). The matrix of the non-gametic phase disequilibria ($\mathbf{L}_0^{''}$) is then given by $\mathbf{L}_0 - \mathbf{L}_0^{'}$ allowing us to compute $\tilde{\mathbf{L}}_0$ as a weighted sum of $\mathbf{L}_0^{'}$ and $\mathbf{L}_0^{''}$, with the the ij^{th} element of the latter being weighted by $r_{ij}/(1 - r_{ij})$, where r_{ij} is the probability of recombination between the two loci. If phased genomes are unavailable, $\tilde{\mathbf{L}}_0$ may be set to $\mathbf{L}_0$ which assumes that the non-gametic phase disequilibria are negligible.

Step 2: Compute the predicted L in generation t in replicate m ($\mathcal{L}_{t,m}$)

Given $\tilde{\mathbf{L}}_0$ in the base population, the expected $\mathbf{L}$ in generation t in replicate m under the action of drift and recombination is given by $\mathcal{L}_{t,m} = \mathbf{N}_{t,m} \circ \tilde{\mathbf{L}}_0$, where the ij^{th} element of $\mathbf{N}_{t,m}$ is $(1 - r_{i,j})^t \prod_{k=0}^{t-1}(1 - \frac{1}{2N_{e_{k,m}}})$, and $\circ$ indicates Hadamard (i.e. element-wise) product. We then sum the $\mathcal{L}_{t,m}$'s for all the generations over which $\Delta\mathbf{p}_m$ is measured to obtain $\mathcal{L}_m = \sum_{t=t_m}^{\tau_m-1} \mathcal{L}_{t,m}$. Note that the expected predicable change due to selection between generations t and τ is $E[\Delta\mathbf{p}_m] = \mathcal{L}_m \boldsymbol{\mu}_{\tilde{\alpha}}$ and the among-replicate covariance in allele frequency change is $COV(\Delta\mathbf{p}_m, \Delta\mathbf{p}_n^\top) = \mathcal{L}_m \mathbf{V}_{\tilde{\alpha}} \mathcal{L}_n$.

Step 3: Compute drift covariance and the projection matrix

Instead of working with raw allele frequency changes, we work with *projected* allele frequency changes. The projection matrix $\mathbf{P}$ is chosen to be such that (1) the number of dimensions of the dataset are reduced to the minimum of N_0 and n_L (typically $N_0 << n_L$), and (2) the allele frequency changes due to drift are independent and identically distributed. First, we perform eigen-decomposition of $\mathbf{L}_0$ (or, equivalently, singular-value decomposition of $\mathbf{C_0}$) and store the eigenvectors corresponding to non-zero eigenvalues ($\mathbf{U_L}$). The (co)variance in allele frequency change due to drift in replicate m is $\tilde{\mathbf{L}}_0 \circ \mathbf{M}^{(m)}$ where $\mathbf{M}^{(m)} = \sum_{t=t_m}^{\tau_m-1} \mathbf{M}_{t,m} \circ \mathbf{N}_{t,m}$ and the ij^{th} element of $\mathbf{M}_{t,m}$ is $(1-r_{i,j})/N_{E_{t,m}}$. We then perform eigen-decomposition of the drift covariance expressed using the eigen-vectors of $\mathbf{L}_0$, i.e. $\mathbf{U}_\mathbf{L}^\top(\tilde{\mathbf{L}}_0 \circ \mathbf{M}^{(m)})\mathbf{U_L}$. Let $\mathbf{U}_2$ be a matrix having the eigenvectors of this $\mathbf{U}_\mathbf{L}^\top(\tilde{\mathbf{L}}_0 \circ \mathbf{M}^{(m)})\mathbf{U_L}$ as columns, and let $\mathbf{D}_2$ be a diagonal matrix of square-rooted eigenvalues of this $\mathbf{U}_\mathbf{L}^\top(\tilde{\mathbf{L}}_0 \circ \mathbf{M}^{(m)})\mathbf{U_L}$. We can then calculate the projection matrix $\mathbf{P} = \mathbf{D}_2^{-1}\mathbf{U}_2^\top\mathbf{U}_\mathbf{L}^\top$, and obtain the projected allele frequency change vector for replicate m as $\Delta\overrightarrow{\mathbf{p}}_m = \mathbf{P}\Delta\mathbf{p}_m$. Note that the mean and covariance of the projected allele frequency changes due to predictable selection are $E[\Delta\overrightarrow{\mathbf{p}}_m] = \mathbf{P}_m\boldsymbol{\mathcal{L}}_m\boldsymbol{\mu}_{\tilde{\alpha}}$ and $COV(\Delta\overrightarrow{\mathbf{p}}_m, \Delta\overrightarrow{\mathbf{p}}_n) = \mathbf{P}_m\boldsymbol{\mathcal{L}}_m\mathbf{V}_{\tilde{\alpha}}\boldsymbol{\mathcal{L}}_n\mathbf{P}_n^\top$.
**Step 4: Preparing data for model**<br>Next, using our models $\boldsymbol{\mu}_{\tilde{\alpha}} = \beta_{\tilde{\alpha}}^{(1)}(\mathbf{p}_0 - \mathbf{q}_0)$ and $\mathbf{V}_{\tilde{\alpha}} = \sigma_{\tilde{\alpha}}^2\mathbf{L}_0^{p_{\tilde{\alpha}}}$ we calculate the fixed effect covariate $\mathbf{P}\boldsymbol{\mathcal{L}}_m(\mathbf{p}_0 - \mathbf{q}_0)$, and the covariance structure of the locus effects as $\mathbf{V}_{m,n} \propto \mathbf{P}\boldsymbol{\mathcal{L}}_m\mathbf{L}_0^{p_{\tilde{\alpha}}}\boldsymbol{\mathcal{L}}_n\mathbf{P}^\top$.
**Step 5: Fit linear mixed models**<br>For a given $p_{\tilde{\alpha}}$, our goals is to fit a linear mixed model with the projected allele frequency change in all replicates ($\Delta\overrightarrow{\mathbf{p}}$) as the response variable and a fixed effect predictor given by $\mathbf{P}\boldsymbol{\mathcal{L}}_m(\mathbf{p}_0 - \mathbf{q}_0)$ with a coefficient $\beta^{(1)}$ to be estimated. We treat locus effects to be random with a covariance structure assumed proportional to $\mathbf{V}_{m,n} \propto \mathbf{P}\boldsymbol{\mathcal{L}}_m\mathbf{L}_0^{p_{\tilde{\alpha}}}\boldsymbol{\mathcal{L}}_n\mathbf{P}^\top$, with a proportionality constant $\sigma_{\tilde{\alpha}}^2$ to be estimated. We use the R function *'optim()'* to find $\widehat{p_{\tilde{\alpha}}}$ that maximises the conditional likelihood of the above model. We then use $\widehat{p_{\tilde{\alpha}}}$, fit the above model, and obtain $\widehat{\beta^{(1)}}$ and $\widehat{\sigma_{\tilde{\alpha}}^2}$.
**Step 6: Estimate** $E[V_{\tilde{A}}(0)]$<br>The final step is to use the estimates obtained in the previous step ($\widehat{p_{\tilde{\alpha}}}$, $\widehat{\beta^{(1)}}$, and $\widehat{\sigma_{\tilde{\alpha}}^2}$) to obtain $E[\widehat{V_{\tilde{A}}(0)}] = Tr(\widehat{\mathbf{L}_0\mathbf{V}_{\tilde{\alpha}}}) + \widehat{\boldsymbol{\mu}_{\tilde{\alpha}}^\top\mathbf{L}_0\boldsymbol{\mu}_{\tilde{\alpha}}}$. We obtain $Tr(\widehat{\mathbf{L}_0\mathbf{V}_{\tilde{\alpha}}})$ as $\widehat{\sigma_{\tilde{\alpha}}^2}Tr(\mathbf{L}_0\mathbf{L}_0^{\widehat{p_{\tilde{\alpha}}}})$, and bias corrected $\boldsymbol{\mu}_{\tilde{\alpha}}^\top\mathbf{L}_0\boldsymbol{\mu}_{\tilde{\alpha}}$ as $\widehat{\beta^{(1)}}^2(\mathbf{p}_0 - \mathbf{q}_0)^\top\mathbf{L}_0(\mathbf{p}_0 - \mathbf{q}_0) - VAR(\widehat{\beta^{(1)}})(\mathbf{p}_0 - \mathbf{q}_0)^\top\mathbf{L}_0(\mathbf{p}_0 - \mathbf{q}_0)$.

S10. Supplementary figures

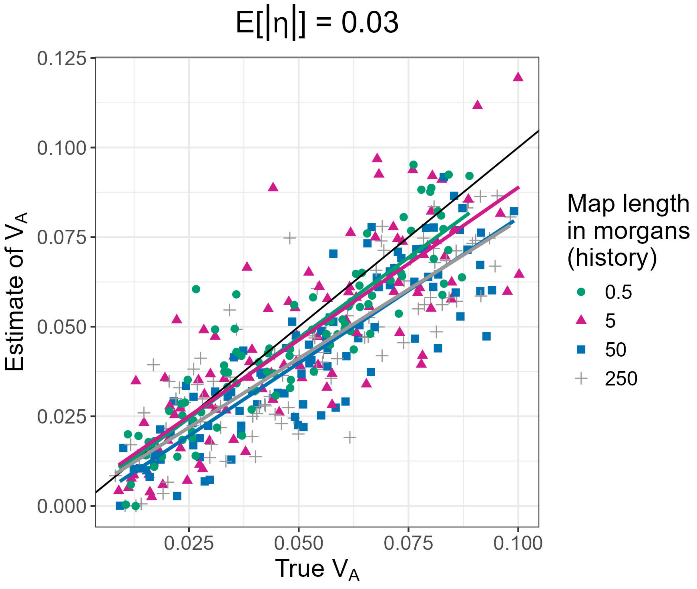

Supplementary Figure 1 Results of full simulations with a burn-in phase of 25,000 generations (map length in the history phase = 2 morgans, number of replicate populations = 10, population size = 1,000, number of generations = 3, the mean of the gamma distribution from which effect sizes for log absolute fitness are sampled for non-neutral mutations ($E[|\eta|]$) = 0.03, at different levels of the map length in the history phase (0.5 morgan: green circles, 5 morgan: magenta triangles, 50 morgans: blue squares, and 250 morgans: grey plus symbols). The coloured solid lines represent regression lines for estimates of V_A vs true values of V_A.

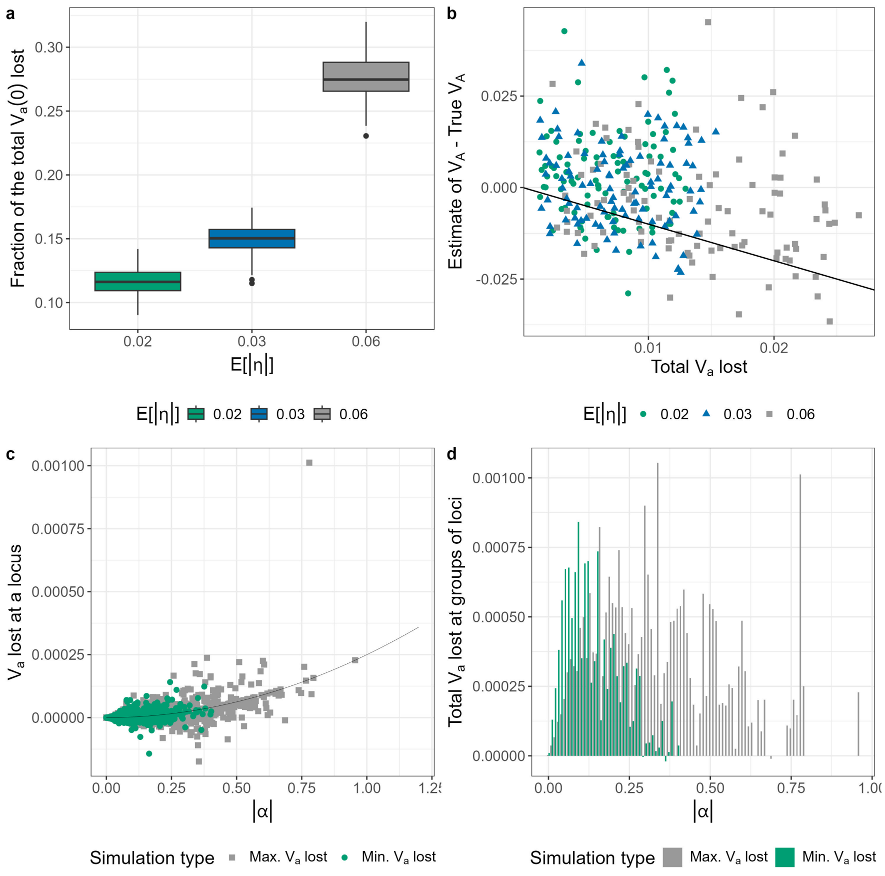

Supplementary Figure 2 (a) Fraction of the total additive genic variance ($V_{\tilde{a}}(0)$) lost during the experiment (averaged over all the replicate populations) plotted as a function of the mean of the gamma distribution from which effect sizes for log absolute fitness are sampled for non-neutral mutations ($E[|\eta|]$). Each boxplot represents 100 independent simulations (for the corresponding level of $E[|\eta|]$) shown in Figure 5D. (b) The error in our estimates of V_A plotted versus the total additive genic variance (V_a) lost during the experiment (averaged over all the replicate populations) for simulations where the $E[|\eta|]$ was either 0.02 (green circles), 0.03 (blue triangles), or 0.06 (grey squares). The solid line has an intercept of 0 and a slope of -1. (c) The contribution of each non-neutral locus (averaged over all the replicate populations) to V_a lost during the experiment plotted versus the absolute average effect for relative fitness ($|\alpha|$) for that locus for a simulation with $E[|\eta|] = 0.06$ that had the maximum V_a loss (grey squares) and a simulation with $E[|\eta|] = 0.02$ that had a comparable true initial V_A but the minimum V_a loss (green circles) in the experiment. The solid curve represents the additive genic variance lost when a singleton goes extinct. (d) The total V_a lost at groups of loci classified by dividing $|\alpha|$ into intervals of 0.01 for the simulation with the maximum V_a loss (grey) and the simulation with the minimum V_a loss (green) in the experiment. In the simulation with the maximum V_A loss – in which the mean $|\alpha|$ for non-neutral segregating loci was 0.0410 – more than 50% of the total loss in V_a was driven by just 0.96% loci whose $|\alpha|$ was greater than 0.3.

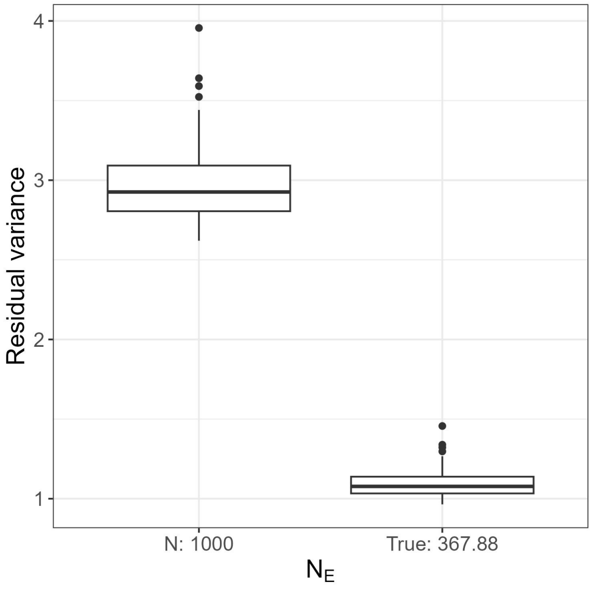

Supplementary Figure 3 Residual variance in the models analysing the results of full simulations shown in Figure 4 using either the number of individuals (1,000) instead of the N_E or the true N_E.

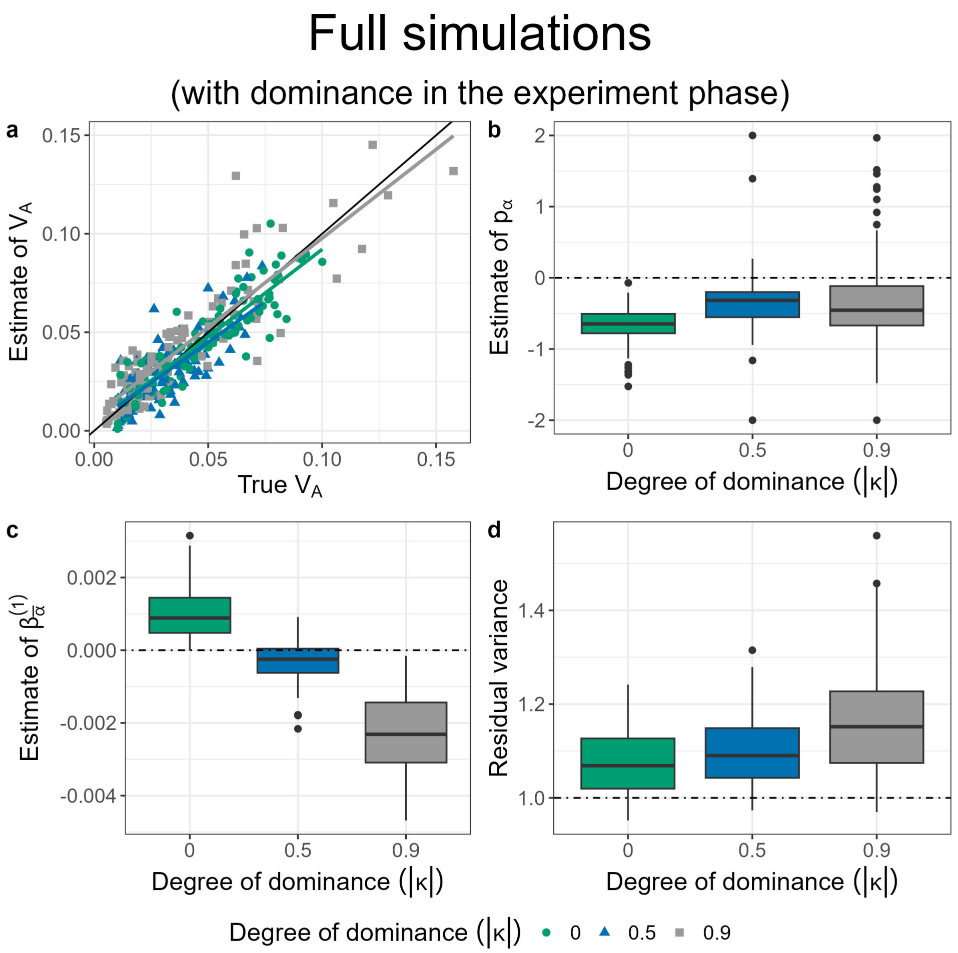

Supplementary Figure 4 Results of full simulations (map lengths in the history phase = 0.5 morgans and experiment phase = 2 morgans, number of replicate populations = 10, population size = 1,000, number of generations = 3, the mean of the gamma distribution from which effect sizes for log absolute fitness are sampled for non-neutral mutations ($E[|\eta|]$) = 0.03), in which dominance effects were switched on only in the experiment phase using different degrees of dominance: $|\kappa| = 0$ (green circles), $|\kappa| = 0.5$ (blue triangles), and $|\kappa| = 0.9$ (grey squares). (a) A scatter plot of estimates of V_A vs true values of V_A. The solid black line indicates the 1:1 line. The coloured lines represent regression lines for estimates of V_A vs true values of V_A. The inference of V_A was obtained by modelling the mean and the (co)variance of the average effects for relative fitness as $\boldsymbol{\mu}_{\bar{\alpha}} = \beta_{\bar{\alpha}}^{(1)}(\mathbf{p}_0 - \mathbf{q}_0)$ and $\mathbf{V}_{\bar{\alpha}} = \sigma_{\bar{\alpha}}^2 \mathbf{L}_0^{p_{\bar{\alpha}}}$, respectively. (b)-(d) Histograms of the estimates of $p_{\bar{\alpha}}$, $\beta_{\bar{\alpha}}^{(1)}$, and the residual variance, respectively. The horizontal dashed lines indicate null expectations (0 for $p_{\bar{\alpha}}$ and $\beta_{\bar{\alpha}}^{(1)}$, and 1 for the residual variance).

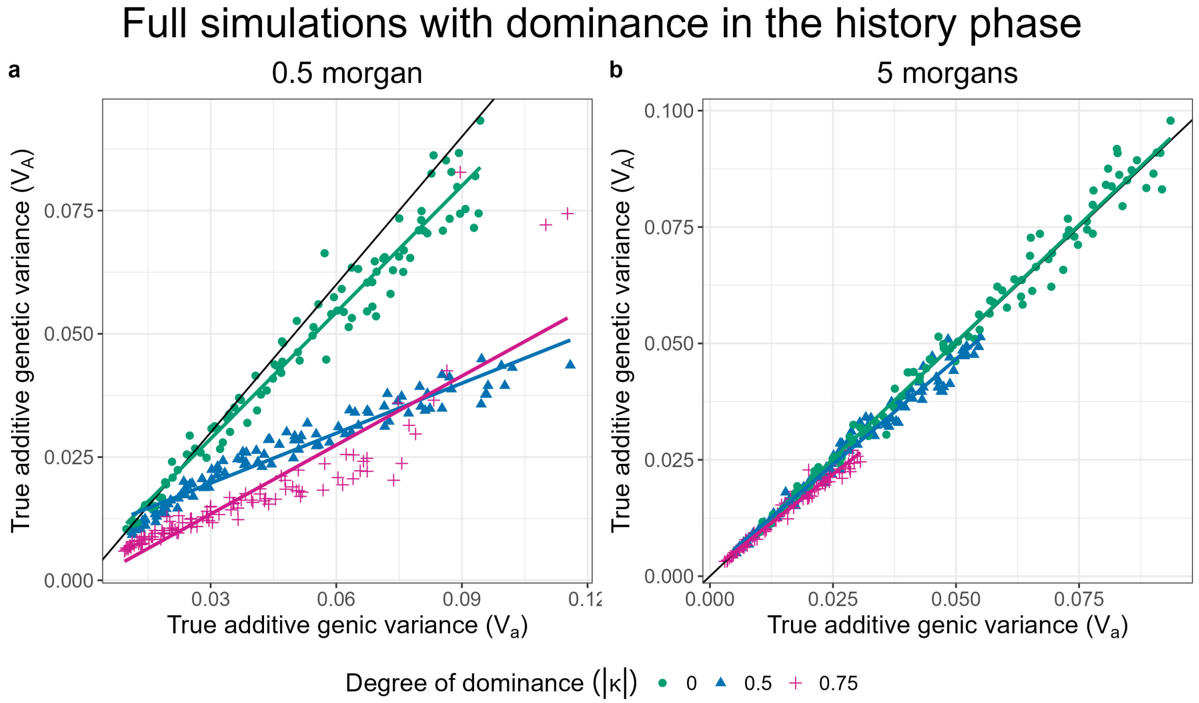

Supplementary Figure 5 The true additive genetic variance for relative fitness (V_A) plotted versus the true additive genic variance for relative fitness (V_a) in full simulations with a burn-in phase of 25,000 generations performed using a map length in the history phase equal to either 0.5 morgan (a) or 5 morgans (b) with dominance effects switched on in the last 5,000 generations of the history phase. Three different degrees of dominance were used: $|\kappa| = 0$ (green circles), $\kappa = 0.5$ (blue triangles), and $\kappa = 0.75$ (magenta plus symbols). The mean of the gamma distribution from which effect sizes for log absolute fitness are sampled for non-neutral mutations ($E[|\eta|]$) was 0.03. The solid black lines indicates the 1:1 line. The coloured lines represent regression lines for true V_A vs true true V_a.

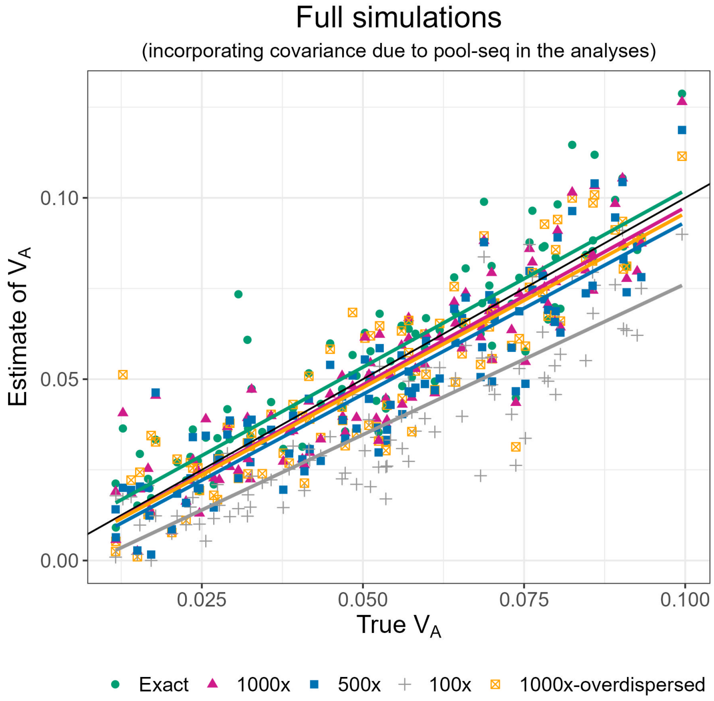

Supplementary Figure 6 Scatter plots of estimates of V_A vs true values of V_A for full simulations with a burn-in phase of 25,000 generations (map length in the history phase = 0.5 morgan, map length in the experiment phase = 2 morgans, number of replicate populations = 10, population size = 1,000, number of generations = 3, the mean of the gamma distribution from which effect sizes for log absolute fitness were sampled for non-neutral mutations ($E[|\eta|]$) = 0.02, and no dominance (i.e. κ = 0)) using either exact allele frequencies in the experiment phase (green circles), or allele frequencies in the experiment phase obtained via simulated pool-seq implemented without any overdispersion in the number of reads mapping to an individual, at three different levels of coverage (expected number of reads mapping a segregating site: 1000x (magenta triangles), 500x (blue squares), and 100x (grey plus symbols)), as well as estimates obtained from simulated pool-seq implemented with overdispersion ($V_x = log(2)$) in the number of reads mapping to an individual, at 1000x coverage (orange empty boxes with crosses). Reads were modelled to be 37 base-pairs long. The solid black line indicates the 1:1 line. The coloured lines represent regression lines for estimates of V_A vs true values of V_A. Estimates of V_A were obtained by incorporating the expected covariance structure due to pool-seq sampling in the models.

Literature cited

Bohrnstedt, G. W., and A. S. Goldberger, 1969 On exact covariance of products of random variables. Journal of the American Statistical Association **64**: 1439–1442.

Buffalo, V., and G. Coop, 2019 The linked selection signature of rapid adaptation in temporal genomic data. Genetics **213**: 1007–1045.

Buffalo, V., and G. Coop, 2020 Estimating the genome-wide contribution of selection to temporal allele frequency change. Proceedings of the National Academy of Sciences **117**: 20672–20680.

Bulmer, M. G., 1980 *The mathematical theory of quantitative genetics.*. Clarendon Press.

de Los Campos, G., D. Sorensen, and D. Gianola, 2015 Genomic heritability: what is it? PLoS Genetics **11**: e1005048.

Ethier, S. N., and T. Nagylaki, 1980 Diffusion approximations of Markov chains with two time scales and applications to population genetics. Advances in Applied Probability **12**: 14–49.

Falconer, D., and T. Mackay, 1996 *Introduction to Quantitative Genetics, 4th edn*. Addison Wesley Longman, Harlow.

Gianola, D., G. de Los Campos, W. G. Hill, E. Manfredi, and R. Fernando, 2009 Additive genetic variability and the Bayesian alphabet. Genetics **183**: 347–363.

Good, B. H., 2022 Linkage disequilibrium between rare mutations. Genetics **220**: iyac004.

Haldane, J. B. S., 1919 The combination of linkage values, and the calculation of distances between the loci of linked factors. Journal of Genetics **8**: 299–309.

Hill, W., and A. Robertson, 1968 Linkage disequilibrium in finite populations. Theoretical and applied genetics **38**: 226–231.

Kojima, K., 1959 Role of epistasis and overdominance in stability of equilibria with selection. Proceedings of the National Academy of Sciences **45**: 984–989.

Lynch, M., and B. Walsh, 1998 *Genetics and analysis of quantitative traits*. Sinauer Associates, Inc., Sunderland.

McCullagh, P., and J. A. Nelder, 1989 *Generalised Linear Models*. Monographs on statistics and applied probability. Chapman & Hall, Cambridge, 2nd edition.

McVean, G. A., 2002 A genealogical interpretation of linkage disequilibrium. Genetics **162**: 987–991.

Meuwissen, T. H., B. J. Hayes, and M. Goddard, 2001 Prediction of total genetic value using genome-wide dense marker maps. genetics **157**: 1819–1829.

Ohta, T., and M. Kimura, 1969 Linkage disequilibrium due to random genetic drift. Genetics Research **13**: 47–55.

Ohta, T., and M. Kimura, 1971 Linkage disequilibrium between two segregating nucleotide sites under the steady flux of mutations in a finite population. Genetics **68**: 571–580.

Santiago, E., and A. Caballero, 1998 Effective size and polymorphism of linked neutral loci in populations under directional selection. Genetics **149**: 2105–2117.

Song, Y. S., and J. S. Song, 2007 Analytic computation of the expectation of the linkage disequilibrium coefficient r^2. Theoretical population biology **71**: 49–60.

Sved, J. A., and W. G. Hill, 2018 One hundred years of linkage disequilibrium. Genetics **209**: 629–636.

Weir, B. S., 1996 *Genetic data analysis II: methods for discrete population genetic data*. Sunderland.

Weir, B. S., and C. C. Cockerham, 1989 Complete characterization of disequilibrium at two loci. In M. Feldman, editor, *Mathematical Evolutionary Theory*. Princeton University Press Princeton, NJ, 86–110.

Wright, S., 1938 Size of population and breeding structure in relation to evolution. Science **87**: 340–431.

Zeng, J., R. De Vlaming, Y. Wu, M. R. Robinson, L. R. Lloyd-Jones *et al.*, 2018 Signatures of negative selection in the genetic architecture of human complex traits. Nature genetics **50**: 746–753.
